# Automated oil spill detection using deep learning and SAR satellite data for the northern entrance of the Suez Canal

**DOI:** 10.1038/s41598-025-03028-1

**Published:** 2025-06-20

**Authors:** Mohamed Zakzouk, Abdulaziz M. Abdulaziz, Islam Abou El-Magd, Abdel Sattar Dahab, Elham M. Ali

**Affiliations:** 1 Mining, Petroleum, and Metallurgical Engineering Department, Faculty of Engineering, Cairo University, Giza, Egypt; 2 Environment Division, National Authority for Remote Sensing and Space Sciences, Cairo, Egypt; 3 Aquatic Environment Department, Faculty of Fish Resources, Suez University, Suez, Egypt

**Keywords:** Oil spill detection, Suez Canal northern entrance, Deep learning, Sentinel-1, SAR imagery, Marine environmental monitoring, Environmental sciences, Ocean sciences

## Abstract

Oil spills threaten marine ecosystems, demanding swift detection and response. The northern entrance of the Suez Canal, a critical maritime route, is increasingly at risk of frequent oil spill incidents. This study employs the DeepLabv3 + deep learning model to automatically detect oil spills in the study area based on Sentinel-1 Synthetic Aperture Radar imagery provided by the European Space Agency. The model was trained separately on two datasets: the European Maritime Safety Agency CleanSeaNet (EMSA-CSN) dataset, comprising 1100 oil spill incidents, and a localized dataset containing 1500 oil spill incidents that occurred at the Egyptian territorial waters. A comparative analysis between the two models was conducted using 30 oil spill test cases located within the study area. The model trained on Egyptian data outperformed the EMSA-CSN-data- trained model, achieving a loss of 0.0516, an accuracy of 98.14%, a mean Intersection over Union (MIoU) of 0.7872, and a significantly higher ROC area of 0.91, compared to a loss of 0.1152, an accuracy of 96.45%, a MIoU of 0.7161, and a ROC area of 0.76 for the EMSA-CSN model. In addition, the area prediction analysis confirmed the superior performance of the Egyptian-data-trained model, which estimated a total affected area of 421.20 km^2^, closely aligning with the ground truth of 425.20 km^2^, whereas the EMSA-CSN-data-trained model underestimated oil spills of around 323.98 km^2^. These results highlight the benefits of region-specific training in improving segmentation quality and reducing errors. This study emphasizes the potential of AI-driven models for real-time oil spill monitoring, with applications in environmental protection and emergency response.

## Introduction

The northern entrance of the Suez Canal faces significant environmental and economic challenges due to its strategic location as a major maritime chokepoint with over 20,000 vessels passing through annually^1^. Oil spills in this region can arise from tanker accidents, operational discharges, or vessel leaks, endangering marine ecosystems, coastal habitats, and local industries such as fisheries and tourism^2^. These spills adversely affect marine life, coating organisms with a toxic layer that impairs their ability to breathe or feed. Spills may also contaminate water resources, posing risks to human health through exposure to toxic chemicals. Furthermore, oil spills near the Suez Canal can disrupt global shipping routes, leading to economic losses for both regional and international trade. Cleanup efforts are complicated by the area’s high traffic and sensitive ecological zones, highlighting the need for advanced detection technologies and coordinated response strategies to mitigate environmental damage^3,4^. Real-time detection and accurate delineation of oil spill extent are critical for effective response and mitigation efforts.

Satellites offer an efficient and cost-effective method for daily scanning of large sea areas to detect potential oil pollution^5^. Multiple remote sensing technologies have been examined for oil spill identification, each offering distinct advantages. Optical remote captures visible and near-infrared light reflected from the water surface, allowing for the identification of thin oil layers under clear conditions, but is hindered by cloud cover and atmospheric interference^6–10^. Thermal infrared imaging detects temperature differences between oil and water, which is useful for identifying thicker oil spills, especially during cooler times or at night^5^. Laser fluorescence uses laser-induced light to excite oil molecules, offering high specificity for oil detection, but is typically limited to smaller-scale or localized applications^11–13^. Multi-angle Imaging SpectroRadiometer (MISR), with its ability to capture multi-angle images, helps in studying the surface roughness and composition of oil spills, providing valuable insights into their size and characteristics^14,15^. Additionally, hyperspectral imaging offers a detailed spectral profile that enables precise characterization of oil spills based on their unique spectral signatures, such as those observed along the Nile River^16^.

Operating in the microwave range, Synthetic Aperture Radar (SAR) is particularly effective for detecting oil spills regardless of weather or illumination conditions, as it captures variations in surface roughness caused by oil, making it ideal for large-scale and all-weather surveillance^17–21^. Although SAR has limitations in precisely quantifying spill thickness, its reliability, wide-area coverage, and adaptability to adverse conditions make it the preferred choice for comprehensive and timely oil spill detection and mapping in this study^22^. Its all-weather and day-night capabilities are indispensable in marine environments, where cloud cover and darkness frequently pose challenges^23,24^.

Detecting oil spills in SAR images is based on its detection mechanism, which leverages the suppression of capillary and short-gravity capillary waves caused by oil spills. This suppression reduces the surface roughness of seawater, appearing as dark spots in SAR images, providing a distinct contrast to the surrounding ocean and enabling effective oil spill detection and monitoring^17^. Effective detection typically requires a minimum wind speed of 2–3 m/s to ensure the oil film is visible; however, high wind speeds larger than 10 m/s can obscure the spill^25^.

However, SAR-based methods face challenges in distinguishing oil spills from other phenomena that also produce dark spots in SAR imagery, such as wave shadows, algal blooms, and low-wind-speed regions behind land masses. These false positives present a significant obstacle to reliable oil spill detection^26^. To overcome this limitation, multi-polarization SAR data offers a solution. By transmitting and receiving signals with varying polarimetric properties, multi-polarization SAR enhances the richness of scattering information, significantly improving the accuracy and reliability of oil spill detection^27^. Consequently, identifying an oil spill in SAR images begins with detecting dark features, which can also include non-oil spill dark signatures resulting from meteorological or oceanographic conditions^4^. The identification procedure typically involves isolating and contouring dark signatures through thresholding, extracting the key parameters related to their shape and radar backscattering contrast. Previous studies on oil spill detection near the entrance of the Suez Canal have primarily employed manual interpretation techniques, as summarized in Table 1^4,21,28–33^.

**Table 1:** Overview of previous oil spill detection studies in the north entrance of the Suez Canal region.

Study reference	Geographical area and period	Utilized satellite data	Detection approach	Manual detection	Number of detected incidents
Abou Samra Ali^28^	Port Said, 2019–2021	Sentinel-1 Sentinel-2	ESA SNAP Toolbox	✓	29
Abou El-Magd et al.^4^	Port Said, 2014–2019	Sentinel-1	ESA SNAP Toolbox	✓	20
Baghdady & Abdelsalam^29^	The Eastern Mediterranean region, 2014–2023	Sentinel-1	ESA SNAP Toolbox	✓	355
Baghdady et al.^30^	Port Said and the northern entrance of the Suez Canal, 2018, 2019	Sentinel-1	ESA SNAP Toolbox	✓	42
Abou El-Magd et al.^21^	The Mediterranean Coast of Egypt, 2014–2020	Sentinel-1	ESA SNAP Toolbox	✓	218
Ferraro et al.^31^	Mediterranean Sea, 1999–2004	ERS-1	Dark feature isolation using thresholding	✓	High oil pollution density
Kostianoy & Lavrova^32^	The Southeastern Mediterranean Sea, 2017–2019	Sentinel-1	Visual Inspection	✓	5
Kostianoy et al.^33^	Mediterranean waters of Egypt, 2017–2019	Envisat, Sentinel-1, Landsat-8	Visual Inspection	✓	28

This manual interpretation of SAR imagery is labour-intensive and susceptible to human error^34^, underscoring the need for automated solutions. Marghany introduced the Quantum Immune Fast Spectral Clustering algorithm for the automatic detection of oil spills in quad-polarized RADARSAT-2 SAR data^35^, leveraging quantum computing principles for feature clustering. This method enhances classification by optimizing feature clustering at a subatomic level, making it highly efficient in detecting subtle differences in oil spill characteristics. However, it relies on quad-polarized RADARSAT-2 data, which is not freely available.

Conversely, deep learning utilizes artificial neural networks to automatically learn patterns and features from large datasets, making it highly effective for complex image analysis tasks like oil spill detection in SAR imagery. By leveraging freely available Sentinel-1 data, the present study provides an accessible and cost-effective solution that ensures efficient and continuous monitoring without the constraints of proprietary data. This approach presents an advancement by utilizing deep learning for automatic oil spill detection in the region.

Numerous Deep learning models have emerged in recent years to address the challenge of oil spill detection from SAR imagery. Krestenitis, M. et al. discussed the use of deep convolutional neural networks (DCNNs) for efficient oil spill detection in SAR images, proposing a new publicly available dataset and demonstrating that DCNNs, particularly DeepLabv3 + , outperform other methods in accuracy and speed^36–38^. Shaban et al. presented a two-stage deep-learning framework for detecting oil spills in SAR images, using a 23-layer CNN for classification and a five-stage U-Net for segmentation, achieving improved precision and dice scores^39^. Singha et al. developed a fully automated approach for detecting oil spills using SAR images, combining classification tree analysis and fuzzy logic, with an accuracy of 85–93% compared to human analysts^40,41^. Keramitsoglou et al. built a fully automated AI-based system for identifying oil spills in SAR images, which was developed and successfully tested on 35 images from the Aegean Sea, providing detailed outputs for decision-making^42^.

Building on this body of research, the present study contributes to this theme by applying semantic segmentation on SAR images for oil spill detection at the northern entrance of the Suez Canal. Additionally, a new dataset was introduced for the scientific community comprising 1500 documented oil spill cases, enabling the model to learn from more localized data and improving its adaptability to regional environmental conditions. This approach enables real-time or near-real-time identification of oil spills, significantly reducing the time and resources needed compared to current manual monitoring methods. Additionally, this framework supports the Egyptian Environmental Affairs Agency (EEAA), which plays a crucial role in reporting and implementing oil spill response plans as part of Egypt’s national contingency strategy for combating marine pollution. This research also aligns with Egypt’s obligations under international conventions, such as MARPOL 73/78 and the Oil Pollution Preparedness, Response, and Co-operation (OPRC ‘90) convention, which require effective measures for environmental monitoring and response efforts.

## Data and methods

### Study area

The geographic region near Port Said and Damietta, situated at the northern entrance of the Suez Canal in northeastern Egypt, has been selected as the study area. This region is crucial to global maritime trade, encompassing the northern exit of the Suez Canal, a vital gateway connecting the Mediterranean Sea to the Red Sea, and facilitating key international shipping routes between Europe and Asia. The area includes the industrial and port cities of Port Said, a major seaport at the canal’s entrance, and Damietta, another important port city located west of Port Said, renowned for its trade and industrial activities. Both cities play pivotal roles in the regional economy and global trade, making the area highly relevant for studying oil spill detection.

**Fig. 1:**
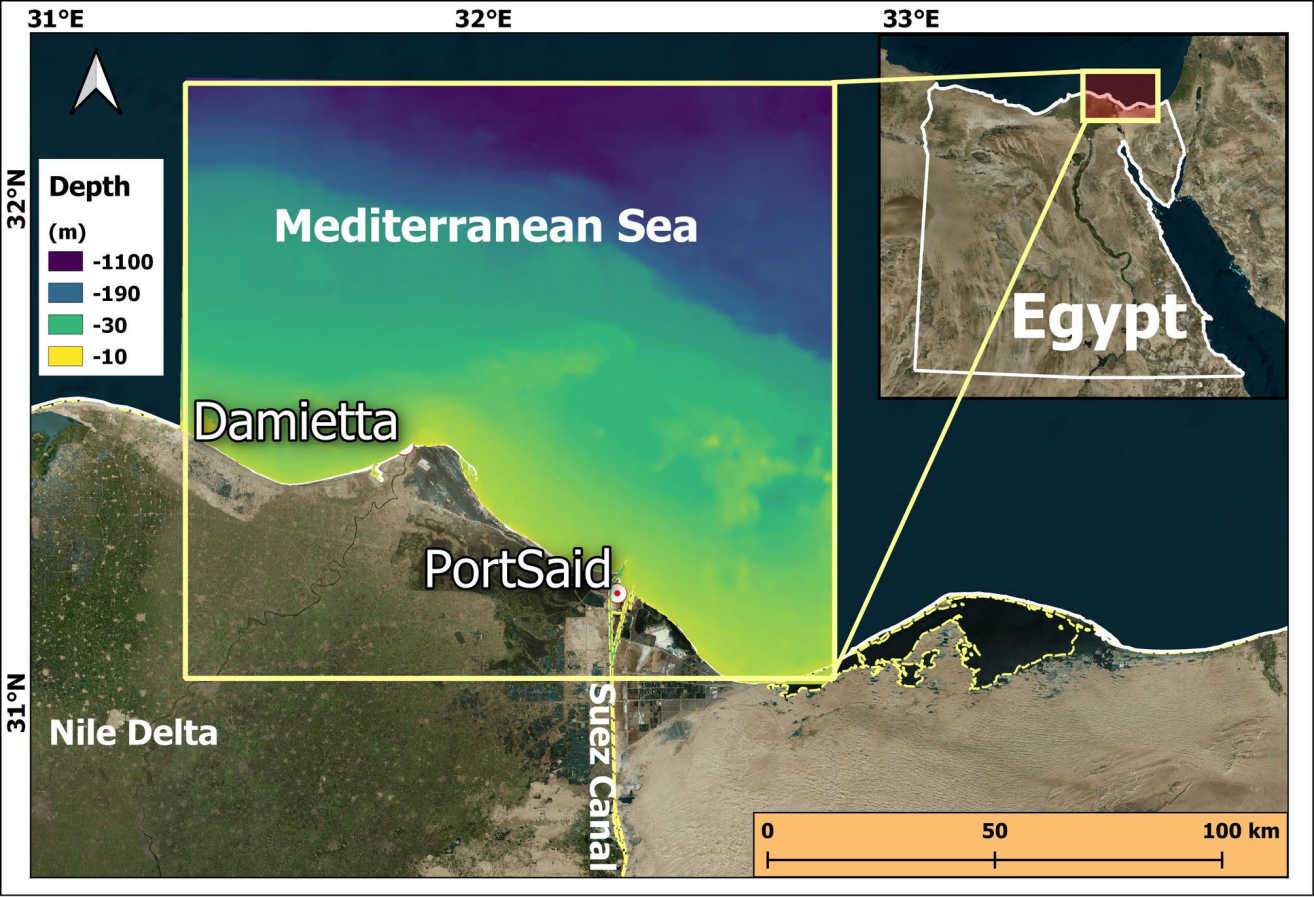
Location of the study area. The map is produced by QGIS software v.3.14.1. https://qgis.org/download/. Bathymetry data was extracted from the National Centers for Environmental Information (NOAA) bathymetry mosaic^43^.

The rectangle defining the area stretches from roughly 31.05°N, 31.33°E in the southwest to 32.25°N, 32.82°E in the northeast. This covers a region of approximately 143 km wide and 131 km long with water depth ranges from -10 to -200 m, with some areas reaching depths of up to -1100 m, as reported by the National Centers for Environmental Information (NOAA) bathymetry mosaic^43^ illustrated in (Fig. 1).

**Fig. 2:**
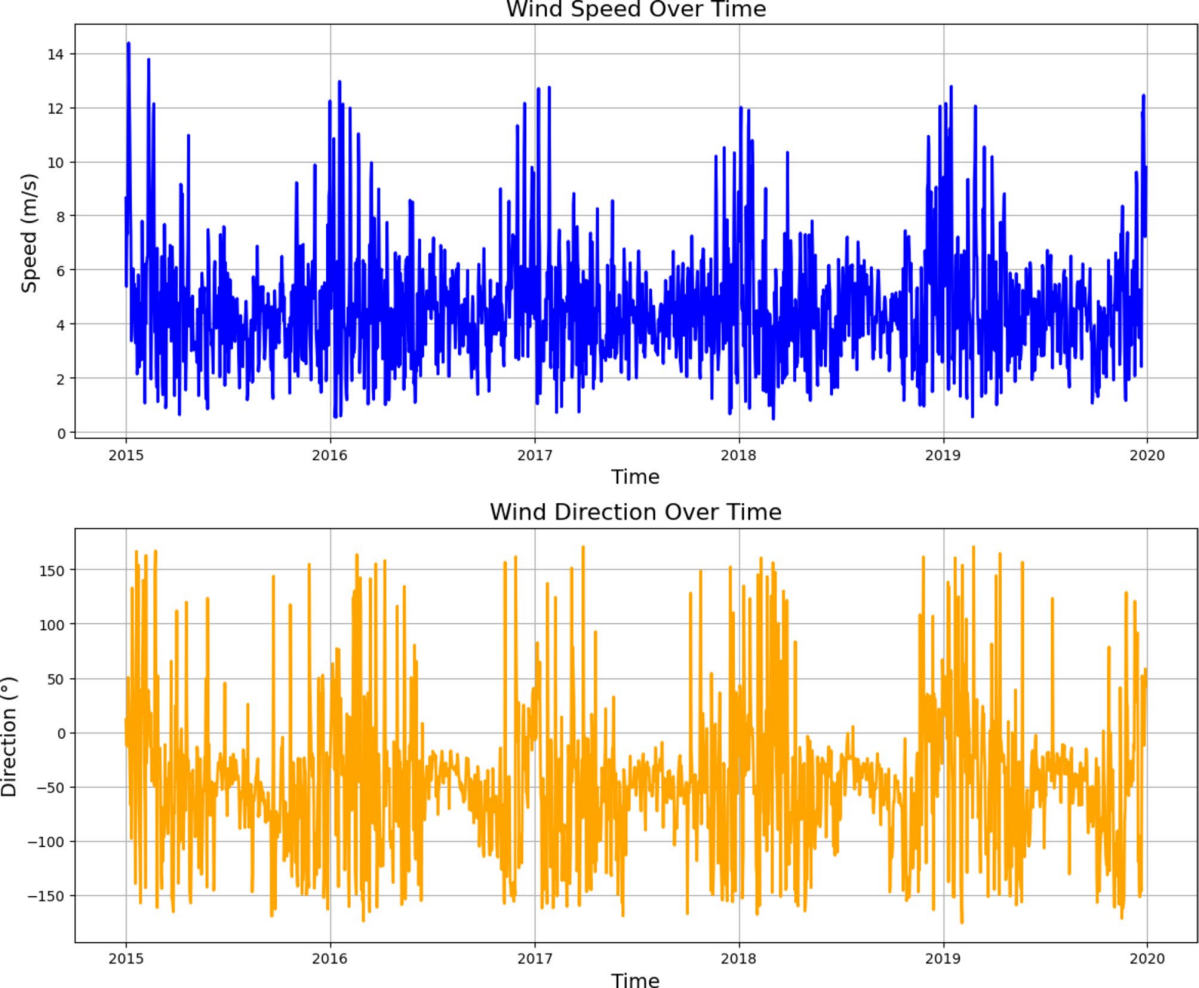
Daily wind speed and direction from 2015 to 2020, extracted from ERA5 daily climate reanalysis modelled data^44^ and visualized using Matplotlib v3.7.1 https://matplotlib.org/.

**Fig. 3:**
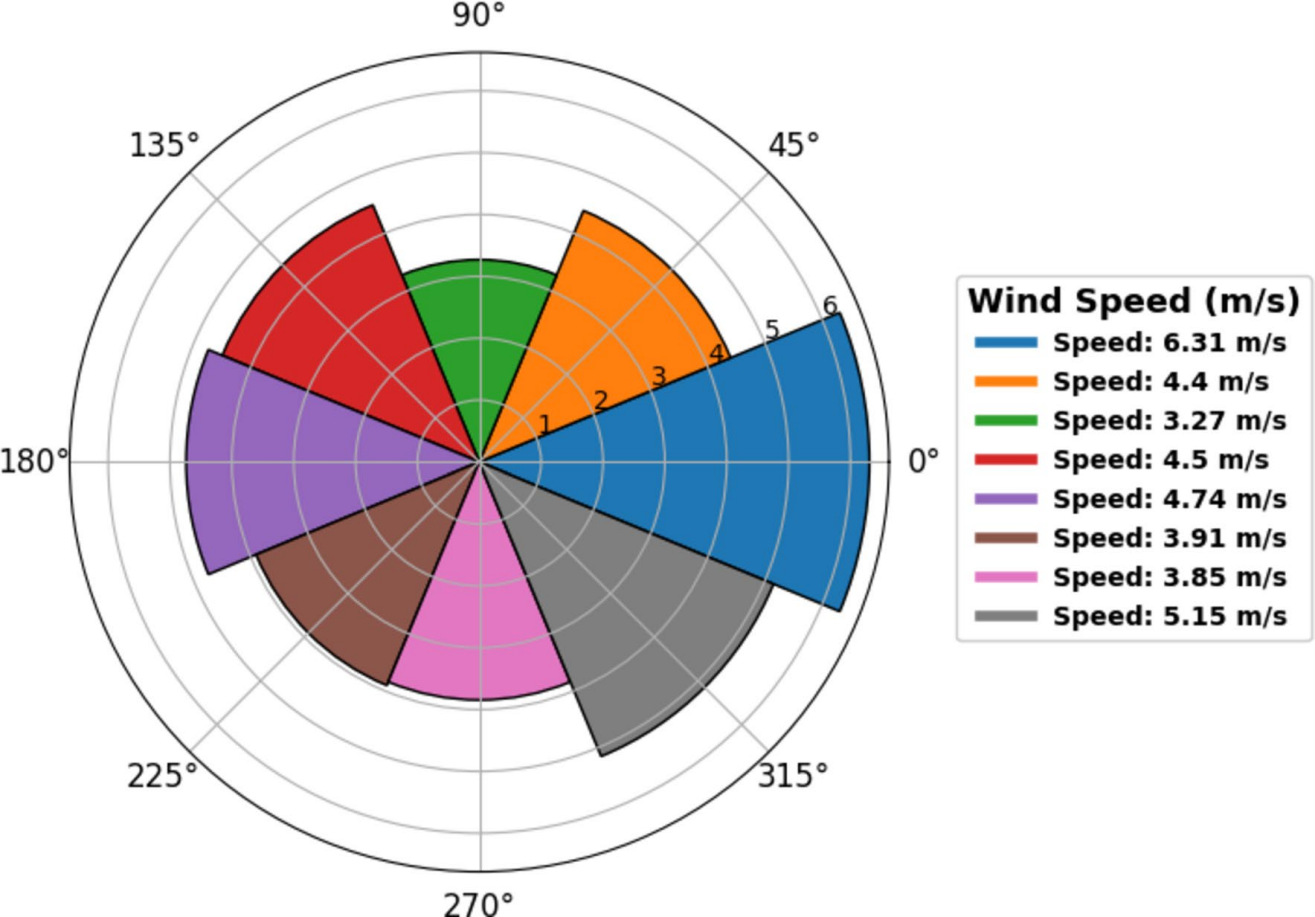
A wind rose diagram illustrating wind speed and direction for the period 2015–2020, extracted from ERA5 daily climate reanalysis modelled data^44^ and plot generated using Windrose v1.9.2. https://cloudsmith.com/navigator/pypi/windrose.

The average wind speed was extracted from ERA5 daily climate reanalysis modelled data produced by the European Centre for Medium-Range Weather Forecasts (ECMWF)^44^. Over the period from 2015 to 2019, wind speed varied between approximately 3 to 6 m/s, which represents favorable conditions for oil spill detection^25^ using Synthetic Aperture Radar (SAR) imagery, as seen in the line charts (Fig. 2) and the wind rose (Fig. 3).

### Dataset

The study utilized Sentinel-1 images provided by the Copernicus Program, a constellation of SAR-equipped satellites launched in 2014 and 2016. It operates in single and dual modes, with a swath width of approximately 250 km in the Interferometric Wide (IW) mode. Operating at the C-band frequency of around 5.405 GHz (wavelength of approximately 5.6 cm), Sentinel-1 provides radar imagery with 10 m spatial resolution^45^. The present study used VV polarization, which enables effective oil spill detection due to the strong contrast between the spill and the surrounding water. Notably, the European Space Agency (ESA) generously provides the Sentinel-1 SAR data product completely free of charge in near-real-time (NRT). ESA provides NRT data delivery for Sentinel-1 within 1–3 h of acquisition, making it a valuable resource for time-sensitive applications such as oil spill detection, maritime safety, and disaster management.

**Fig. 4:**
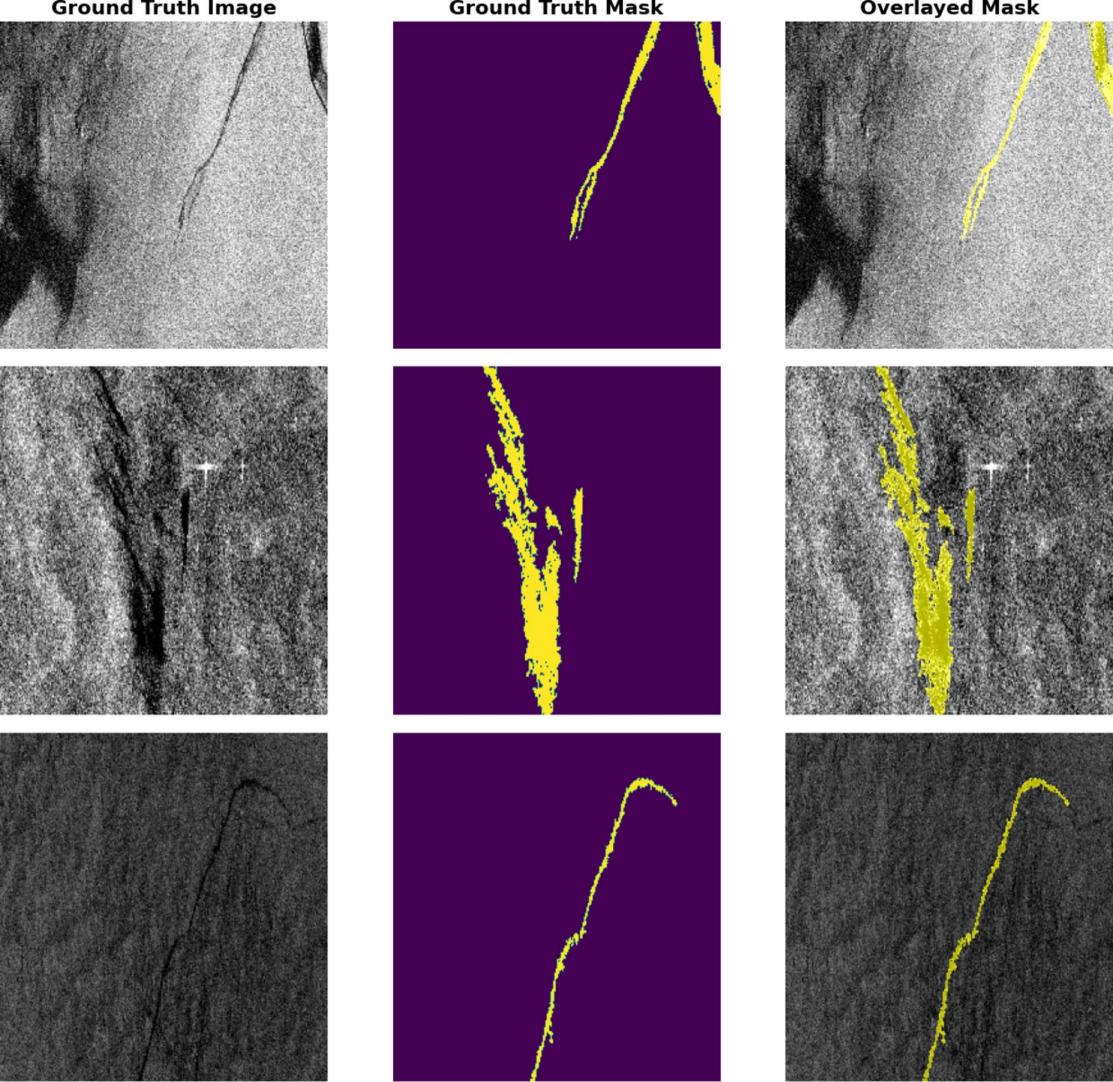
SAR images depict EMSA-CSN oil spills alongside corresponding ground truth masks^37^. Data is prepared by OpenCV-Python v.4.10 https://docs.opencv.org/4.x/ and plotted by Matplotlib v.3.7.1 https://matplotlib.org/.

For model training, the labelled oil spill dataset produced by the European Maritime Safety Agency (EMSA) through the CleanSeaNet (CSN) service^37^ has been utilized. This dataset comprises 1,110 Sentinel-1 SAR imagery saved as 3-channel RGB images, along with expertly annotated masks. Importantly, each polygon in the dataset corresponds to a confirmed oil spill incident that was validated by EMSA analysts through standard post-analysis procedures. These masks are meticulously crafted to delineate digitized oil spills, ships, lookalikes, and the water surface. To simplify the task into a binary classification problem, the data was clipped to focus exclusively on oil and non-oil regions (Fig. 4). While the raw Sentinel-1 SAR imagery is freely available through the Copernicus Open Access Hub, the EMSA-CSN annotated dataset was obtained through direct communication with the authors of the source study for non-commercial academic research under specific data-sharing arrangements, following EMSA’s data access policies.

For robust model training, a 90–10 split ratio between training and validation samples is used for evaluating model performance.

**Fig. 5:**
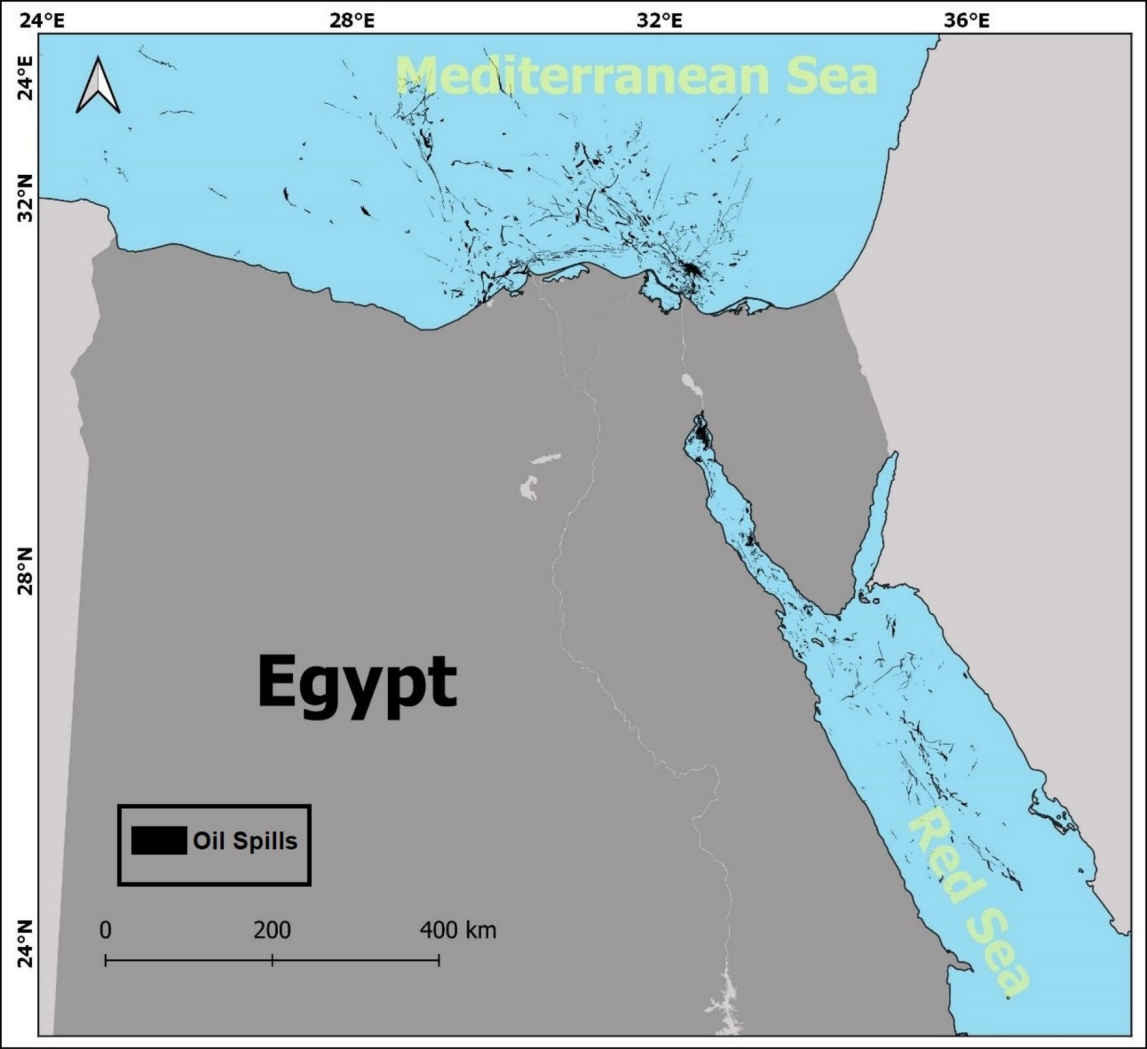
Annotations for 1500 oil spill incidents in the Egyptian seas that were utilized for model training. The map was generated using QGIS software (v.3.14.1) https://qgis.org/download/.

In addition to the EMSA-CSN dataset, a historical dataset comprising approximately 1500 documented oil spills that occurred between 2014 and 2024 in the Egyptian seas, including parts of the Mediterranean Sea^21^ and the Red Sea^3^, has been incorporated. This dataset introduces regional specificity that facilitates a comparative analysis across diverse geographic and operational conditions. Since high-quality labelled data specific to the study area is limited, relevant data from surrounding regions was also incorporated to enhance model training. The dataset covers a total of around 15,000 square kilometers of oil spill area. (Fig. 5) provides a visual representation of all annotated oil spill occurrences.

**Fig. 6:**
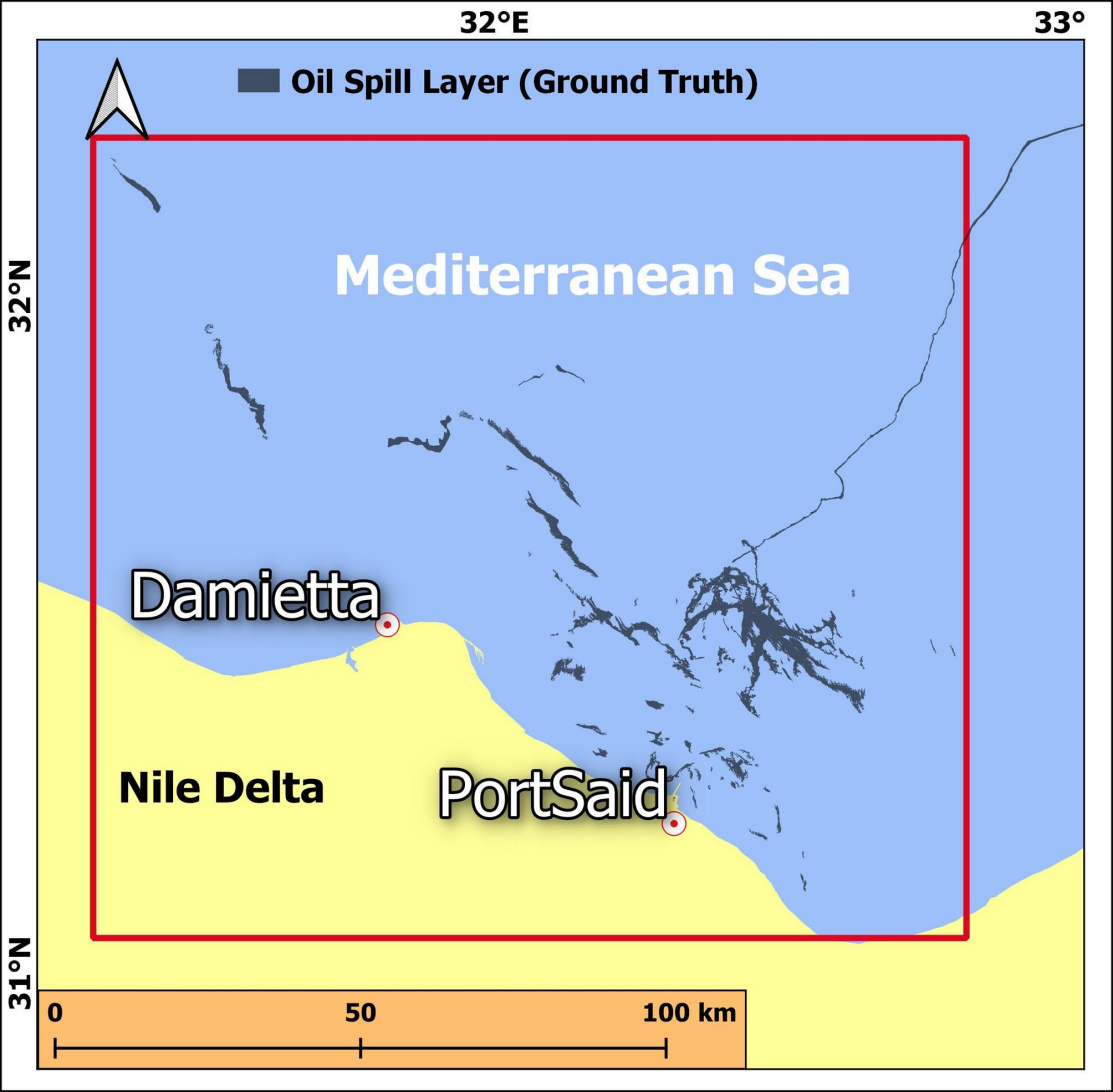
Ground truth testing dataset. The map is produced by QGIS software v.3.14.1. https://qgis.org/download/.

For both models testing, a total of 30 oil spill cases from the study area were carefully selected to assess their performance under the dominant area conditions. These cases represent a diverse range of spill sizes, shapes, and environmental conditions, ensuring a comprehensive evaluation (Fig. 6). The selection process also aimed to include both large and small spills and varying levels of complexity in surrounding features to provide the suitable capacity for accurate detection and sharp delineation of oil spills in different scenarios.

### Methods

#### SAR preprocessing

The preprocessing pipeline for Synthetic Aperture Radar (SAR) images involved a series of critical steps designed to enhance the quality and usability of the data for subsequent analysis. The process begins with orbit file correction, which refines the orbit data to ensure accurate geolocation. This is followed by radiometric calibration, which converts raw radar data digital pixel values into radiometrically calibrated backscatter units (Eq. 1) of consistent measurement across different images^46^.

1 $$\text{Calibrated Value}(\text{i})=\frac{{DN}_{i}^{2}}{{A}_{i}^{2}}$$

where

- DN is the Digital Number value of the pixel.
- ${A}_{i}$ is the calibration factor obtained from the calibration Look-Up Tables (LUTs).

Speckle noise reduction (Eq. 2) is then applied using the Lee Sigma filter (7 ×  7)^47^, to mitigate noise and improve the clarity of the image as follows:

1. Calculate the local mean (μ) and standard deviation (σ) within a 7 × 7 window.
2. For each pixel *I*(*i*,*j*) in the image, the output of the filter *O*(*i*,*j*) is computed as:
  2 $$O\left(i,j\right)=\upmu +\frac{I\left(i,j\right)-\upmu }{{\upsigma }^{2}+\epsilon }\bullet {\upsigma }_{filtered}$$

where

- ${\upsigma }_{filtered}$ is a user-defined or predetermined value set at 0.9, representing the desired standard deviation after filtering.
- ∈ is a small constant to avoid division by zero.

The next step, linear to decibel conversion, transforms the data from a linear scale to decibels, facilitating better interpretation of intensity variations. Geometric correction follows, where orthorectification adjusts the image to correct distortions related to the sensor’s perspective and terrain^48^. Following that, stretching is applied to enhance the contrast of the image, making it more suitable for analysis.

**Fig. 7:**
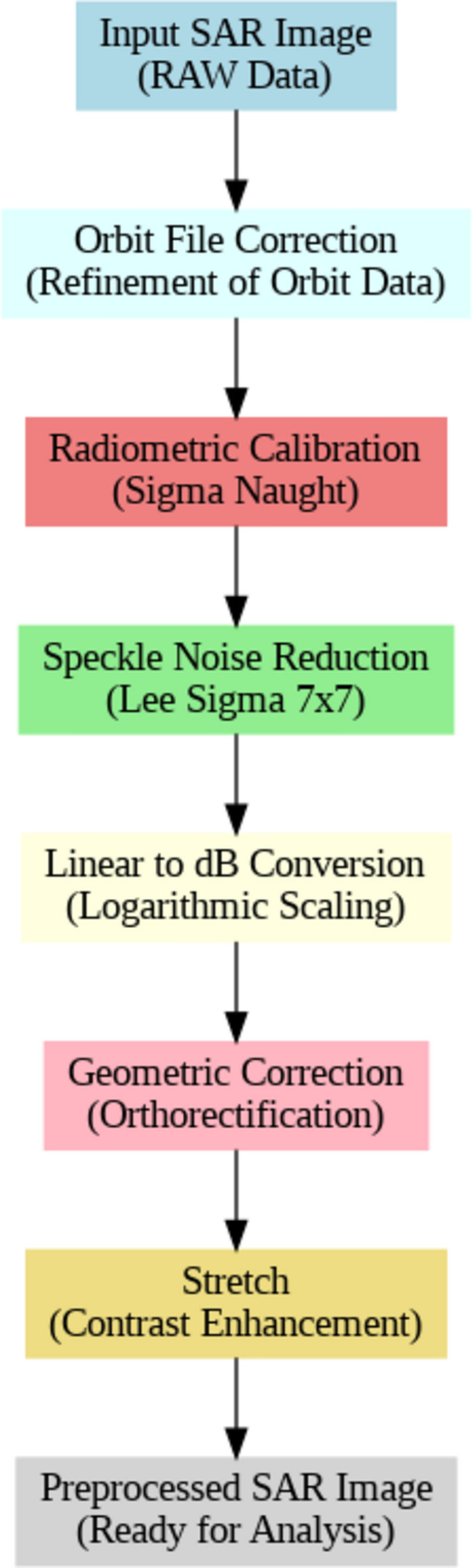
The SAR preprocessing pipeline. The workflow graph is produced by the Graphviz library v.0.20.3 https://graphviz.readthedocs.io/en/stable/.

Finally, the preprocessed GeoTIFF SAR images are converted into a 3-channel RGB image, making it suitable for subsequent training and testing in oil spill detection tasks. This conversion ensures that the grayscale SAR data is transformed into a format compatible with standard deep-learning models while retaining the critical information necessary for accurate oil spill analysis. The pixel values are first normalized to fit within the 0–255 range, ensuring the intensity values are appropriate for RGB display. After normalization, the grayscale values are replicated across the red, green, and blue channels, producing a color representation that preserves the underlying intensity information of the original SAR image. This process facilitates both visualization and integration into deep learning workflows without compromising the data’s analytical value. (Fig. 7) provides a flow chart for the preprocessing pipeline applied to SAR images, preparing them for subsequent analysis and evaluation.

#### Model training and validation

**Fig. 8:**
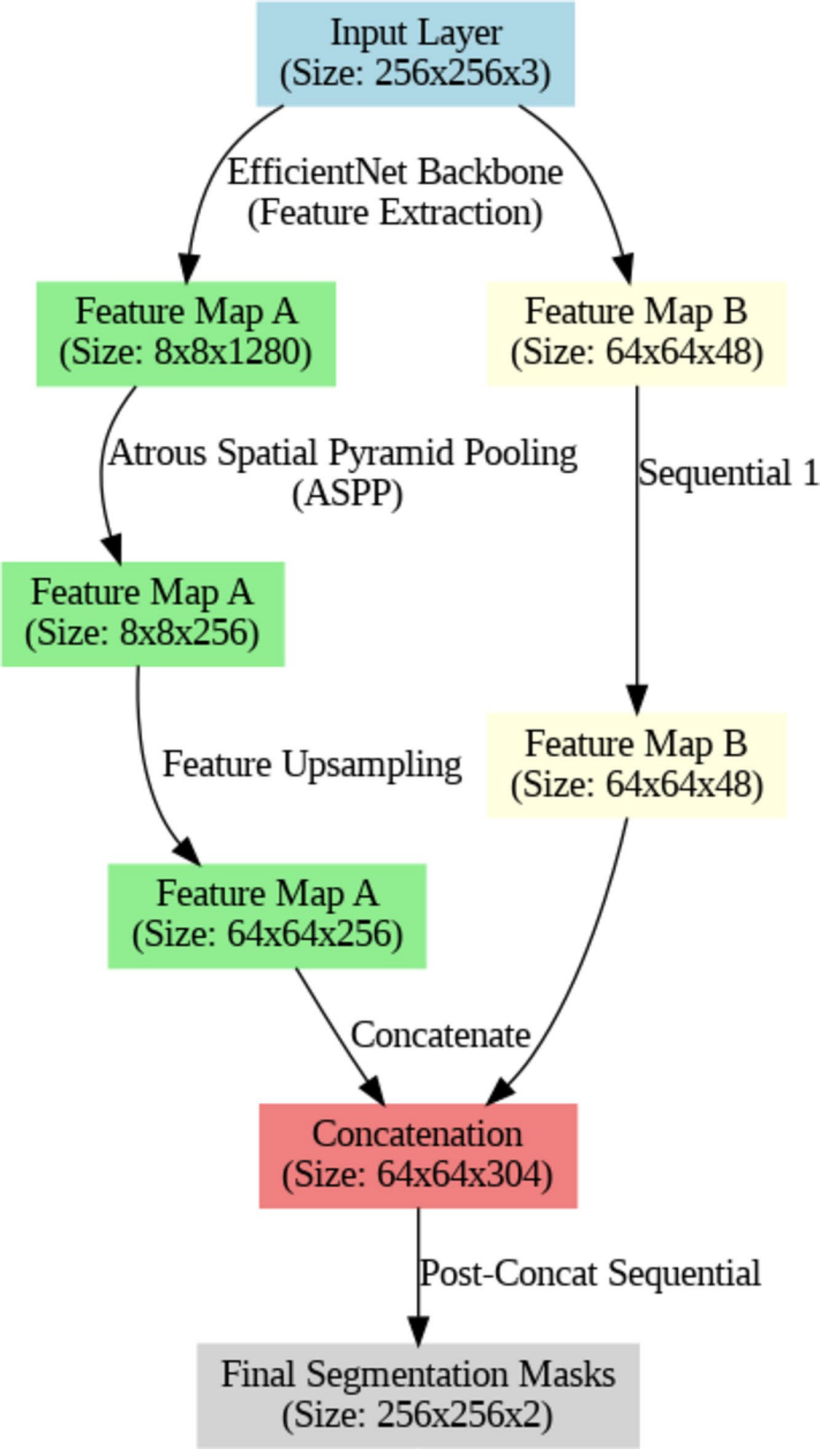
A diagram of the DeepLabv3 + model’s architecture, showing the shapes and names of each layer. The workflow graph is produced by the Graphviz library v.0.20.3 https://graphviz.readthedocs.io/en/stable/.

The DeepLabv3 + architecture was selected for its state-of-the-art performance in semantic segmentation tasks, as designed by Google researchers^49^. This model extends the capabilities of the DeepLabv3 architecture by incorporating an encoder-decoder structure with Atrous Spatial Pyramid Pooling (ASPP) and a Feature Pyramid Network (FPN). The encoder component of DeepLabv3 + typically employs a Convolutional Neural Network (CNN) with an EfficientNet (EffNet) backbone. The EffNet backbone is utilized for its efficiency in extracting high-level features from the input image while balancing accuracy and computational cost. (Fig. 8) shows the DeepLabv3 + model’s architecture.

The model begins with an input layer that accepts images of size 256 × 256 pixels with 3 color channels (RGB) serving as the entry point for image data. The functional layer, which is based on EfficientNet, serves as the core feature extractor. It outputs two sets of feature maps: one with a spatial dimension of 64 × 64 pixels and 256 channels and another with a spatial dimension of 8 × 8 pixels and 2048 channels. This layer is responsible for capturing high-level features from the input images. The spatial pyramid pooling layer applies atrous convolutions at multiple rates to the feature maps from the EfficientNet backbone. It outputs feature maps with a spatial dimension of 8 × 8 pixels and 256 channels. This layer is crucial for capturing multi-scale contextual information. The encoder output upsampling layer upsamples the feature maps to a spatial dimension of 64 × 64 pixels but retains 256 channels. This layer is used to adjust the spatial resolution of the features for the subsequent processing steps. The concatenate layer merges the upsampled feature maps with additional features from the sequential layer. The concatenated output has a spatial dimension of 64 × 64 pixels and 304 channels. This layer facilitates the fusion of features from different sources. The post concatenation sequential layer, which represents the final decoder stage, processes the concatenated features to produce the segmentation masks. It outputs feature maps with a spatial dimension of 256 × 256 pixels and 2 channels, corresponding to the pixel-wise classification of the image. This layer refines the segmentation output to match the original input size.

This architecture balances high performance with computational efficiency, utilizing a mix of complex and efficient layers to achieve accurate segmentation. The EfficientNet backbone and ASPP module are key to capturing multi-scale features and contextual information, while the upsampling and concatenation steps refine the final segmentation masks. The training configuration involved 100 training epochs and a learning rate of 0.0001, which are crucial for the model’s effectiveness and convergence during training.

#### Testing

**Fig. 9:**
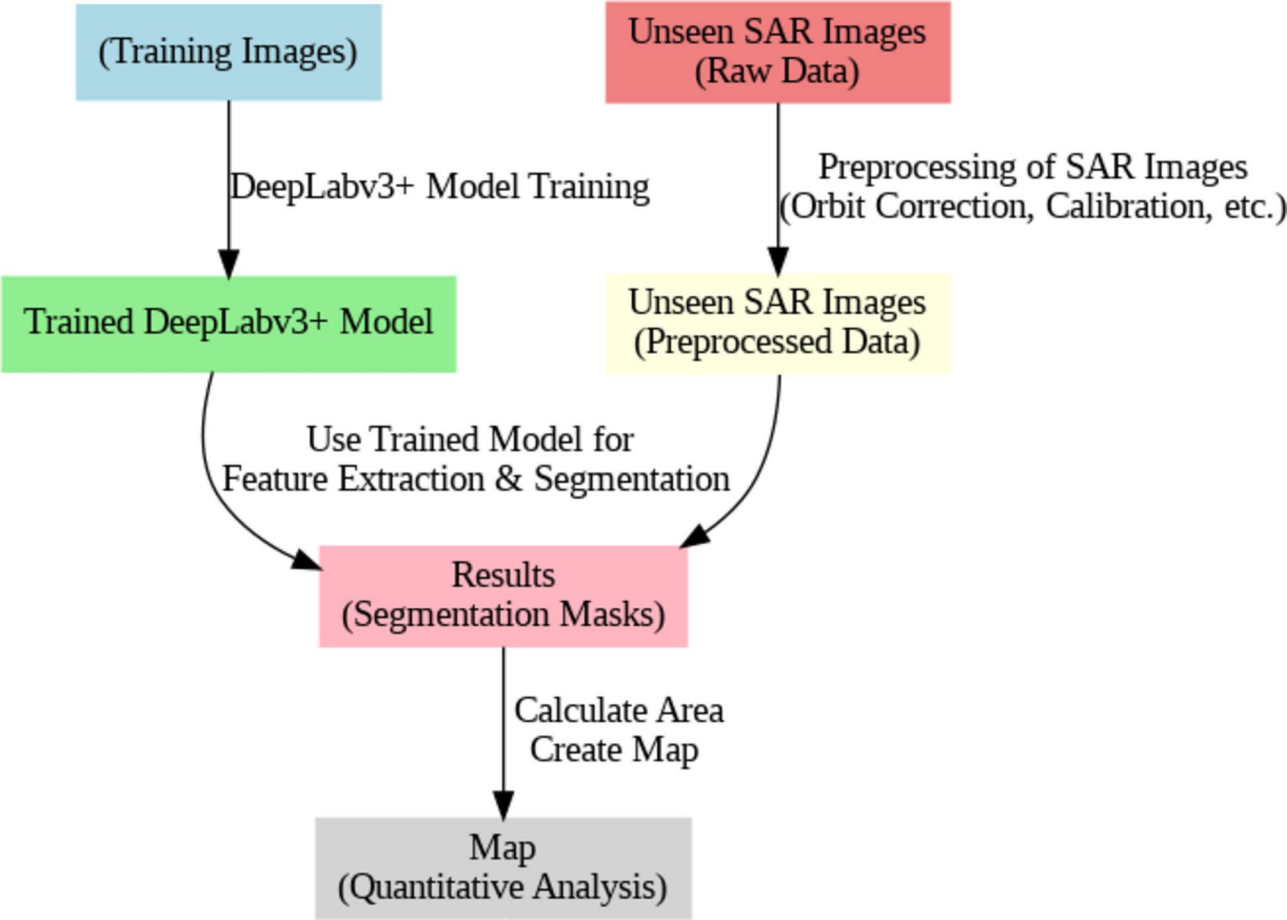
Workflow for testing unseen images from the study area. The workflow graph is produced by the Graphviz library v.0.20.3 https://graphviz.readthedocs.io/en/stable/.

Both trained models are employed to test unseen images from the study area according to the procedure in (Fig. 9). The preprocessed SAR images are fed into the trained model, which produces segmentation masks that identify regions affected by oil spills. These masks are subsequently converted from RGB format back to GeoTIFF images to enable the generation of detailed maps and the execution of quantitative area calculations. This conversion facilitates a comprehensive analysis and visualization of the SAR data that enhances both assessment and interpretation for spatial distribution and extent of oil spills.

The model’s performance on the unseen testing images was assessed using accuracy, loss and mean intersection over union (MIoU) (Eqs. 3–5). Among these, MIoU is a critical metric in segmentation tasks, providing a robust measure of the model’s accuracy in identifying and delineating relevant features within the data. MIoU measures the average overlap between the predicted and ground truth masks to provide a comprehensive assessment of the model’s segmentation accuracy. When presented with two segmentation masks, A and B:

3 $$\text{Accuracy}=\frac{TP+TN}{\text{TP}+\text{TN}+\text{FP}+\text{FN}}$$

4 $$\text{MIoU}=\frac{\text{Intersection}}{\text{Union}}=\frac{|\text{A}\cap \text{B}|}{|\text{A}\cup \text{B}| }=\frac{TP}{\text{TP}+\text{FP}+\text{FN}}$$

where

- True Positive (TP) is the number of pixels correctly predicted as the target class.
- False Positive (*FP*) is the number of pixels incorrectly predicted as the target class.
- False Negative (FN) is the number of pixels that belong to the target class but were not predicted as such.
- True Negative (TN) is the number of instances that are actually negative and are correctly predicted as negative.

The term loss quantifies how well the predicted segmentation masks match the ground truth masks. It can be expressed as:

5 $$Loss=-\frac{1}{N}\sum_{i=1}^{N}[{y}_{i}\text{log}(\widehat{{y}_{i}})+(1-{y}_{i})\text{log}(1-\widehat{{y}_{i}})]$$

where $\widehat{{y}_{i}}$ is the predicted probability of the pixel being in the target class. ${y}_{i}$ is the ground truth pixel value (1 for target class, 0 for background). $N$ is the total number of pixels.

The Receiver Operating Characteristic (ROC) curve and confusion matrix have also been incorporated to evaluate the performance of the models’ results.

ROC curve is a graphical representation used to evaluate the performance of a binary classification model. It plots the True Positive Rate (TPR) against the False Positive Rate (FPR). Area Under the Curve (AUC) quantifies overall model performance. Higher AUC indicates better discrimination between classes.where

6 $$\text{TPR}=\frac{TP}{\text{TP}+\text{FN}}$$

7 $$\text{FPR}=\frac{FP}{\text{FP}+\text{TN}}$$

The confusion matrix provides a summary of prediction results by showing the number of correct and incorrect predictions, categorized by class, as shown in Table 2.

**Table 2:** Confusion matrix overview.

True positive (TP)	False negative (FN)
False Positive (FP)	True Negative (TN)

All assessments were conducted with a prediction threshold of 0.5 for the generated masks, meaning that any pixel classified with a probability greater than or equal to 0.5 was considered a positive detection, while those below this threshold were deemed negative. This binary classification approach ensures that only predicted areas with reasonable confidence contribute to the evaluation metrics.

##### Hardware and software packages

The preprocessing pipeline for the project was developed using ESA’s SNAP GPT Graph Builder (v.6.0), while Google Colab was utilized for both training and testing of the model. Google Colab with NVIDIA Tesla T4 GPU, which features 16 GB of GDDR6 memory and is based on the Turing architecture, is ideal for deep learning tasks. The CPU configuration typically includes 2 vCPUs from an Intel Xeon processor. In terms of memory, Colab Standard offers 12 GB of RAM.

Various Python libraries were used for efficient handling, processing, and analyzing SAR imagery within the pipeline. Pandas (v.2.2.2) is used for data manipulation, offering powerful data structures like DataFrames. For geospatial data, Fiona (v.1.10.1) and Rasterio (v.1.4.1) facilitate reading and writing vector and raster data, respectively, enhancing interaction with geographic information systems (GIS) and supporting formats like GeoTIFF. NumPy (v.1.26.4) serves as the foundational package for numerical computing, supporting large multi-dimensional arrays. Matplotlib (v.3.7.1) is employed for creating visualizations, while OpenCV-Python (v.4.10.0) provides tools for image processing and analysis in real-time computer vision tasks. Graphviz (v.0.20.3) is utilized for generating visual representations of model architectures and other graphical workflows and Contextily (v.1.6.2) enhances map visualizations by providing basemaps for geospatial data plotting. Finally, Scikit-learn (v.1.5.2) offers efficient machine learning tools for data mining and various algorithms for classification, regression, and clustering. To access and download Sentinel satellite imagery, Sentinelsat (v.1.2.1) is employed, allowing for straightforward interaction with the Copernicus Open Access Hub. GeoPandas (v.1.0.1), an extension of Pandas, facilitates working with geospatial data by offering geospatial operations and data structures. The training and evaluation of models are enhanced by TensorBoard (v.2.15.2), a visualization tool for monitoring and analyzing machine learning model performance. To build and train the models, Keras-CV (v.0.9.0) is utilized alongside TensorFlow (v.2.15.0), an open-source machine learning framework that offers comprehensive tools and libraries. Additionally, QGIS (v.3.14.1-Pi) is employed for handling geospatial data visualization and manipulation. Microsoft Office LTSC Professional Plus 2021 supports documentation and data presentation tasks.

The speed at which SAR analyses can be disseminated to field responders depends on the processing workflow, infrastructure, and communication systems in place. Typically, SAR data from Sentinel-1 is available in near-real-time (NRT) within 1–3 h of acquisition. After receiving the data, the automated processing and analysis workflow can detect oil spills or other anomalies in minutes to an hour. Overall, the processed information can be shared with responders within 3–4 h of data acquisition.

## Results and discussion

### Model trained on EMSA-CSN data

**Fig. 10:**
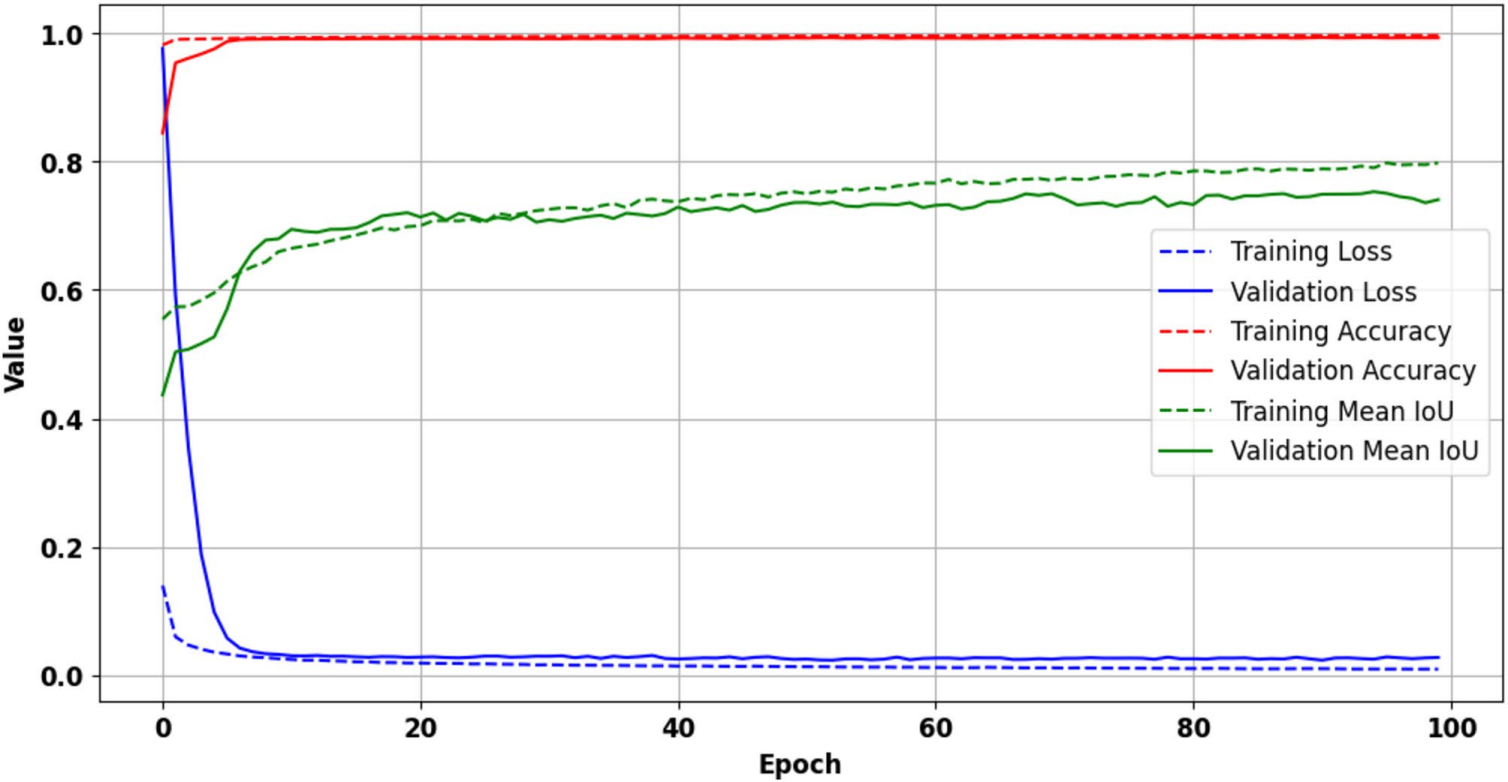
Training and validation metrics with EMSA-CSN data across 100 epochs. The presented data is prepared by TensorBoard library v.2.15.2 https://pypi.org/project/tensorboard/ and plotted by Matplotlib v.3.7.1 https://matplotlib.org/.

The first model trained on EMSA-CSN data performed well in both the training and validation phases. At epoch 100, it achieved a training loss of 0.0093, a training accuracy of 99.65%, and a MIoU of 0.7978. The validation performance achieved a loss of 0.0275, an accuracy of 99.29%, and a MIoU of 0.7407. These metrics, as shown in (Fig. 10), suggest that the model generalizes well to unseen data.

**Fig. 11:**
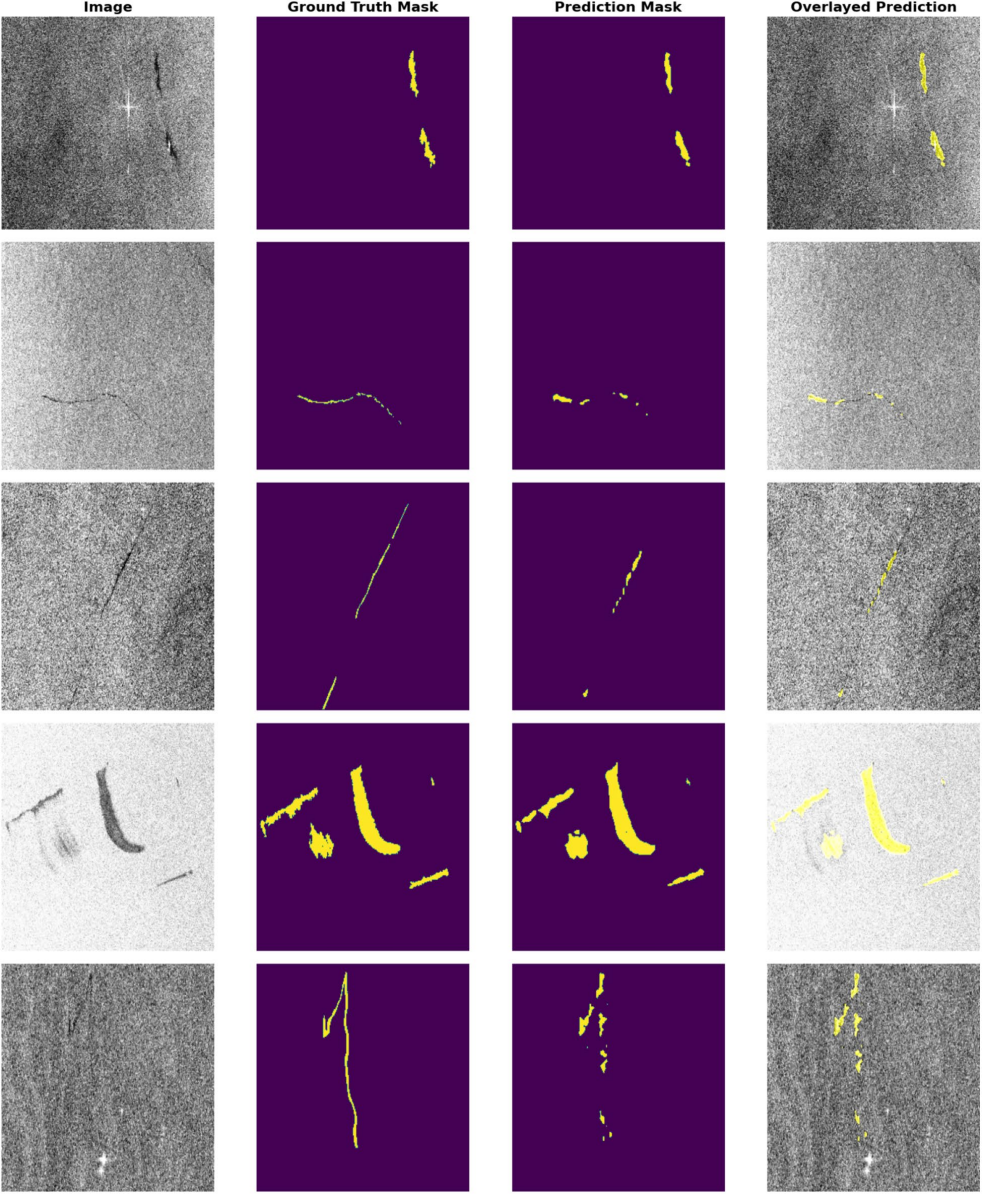
A comparison between predictions generated by the EMSA-CSN-data-trained model on its validation data. The presented data is prepared by Keras-CV library v.0.9.0 https://pypi.org/project/keras-cv/ and plotted by Matplotlib v.3.7.1 https://matplotlib.org/.

In addition to quantitative metrics, the fine-tuned model’s qualitative performance is showcased through its predictions. (Fig. 11) presents these predictions and demonstrates the model’s response in understanding and interpreting visual content within images.

### Model trained on Egyptian dataset

**Fig. 12:**
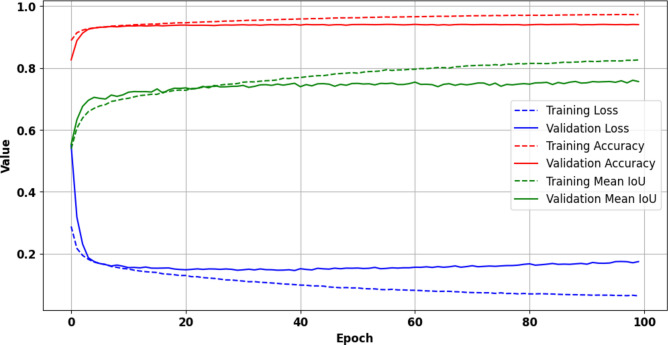
Training and validation metrics over 100 epochs for the Egyptian dataset, visualized demonstrating model performance during the training process. The presented data is prepared by TensorBoard library v.2.15.2 https://pypi.org/project/tensorboard/ and plotted by Matplotlib v.3.7.1 https://matplotlib.org/.

On the other side, the model trained on the Egyptian dataset also demonstrated good results but with slightly different performance characteristics, as shown in (Fig. 12). At epoch 100, the training loss was 0.0599, the accuracy was 97.41%, and the MIoU was 0.8367. Although the accuracy was slightly lower compared to the EMSA-CSN-data-trained model, the MIoU was marginally higher, suggesting that the model trained on the Egyptian dataset was able to delineate oil spill boundaries precisely and capture finer details in the segmentation. On the validation dataset, the model reported a loss of 0.1744, an accuracy of 93.99%, and a MIoU of 0.7559, showing a slightly higher loss and lower accuracy compared to the EMSA-CSN-data-trained model.

**Fig. 13:**
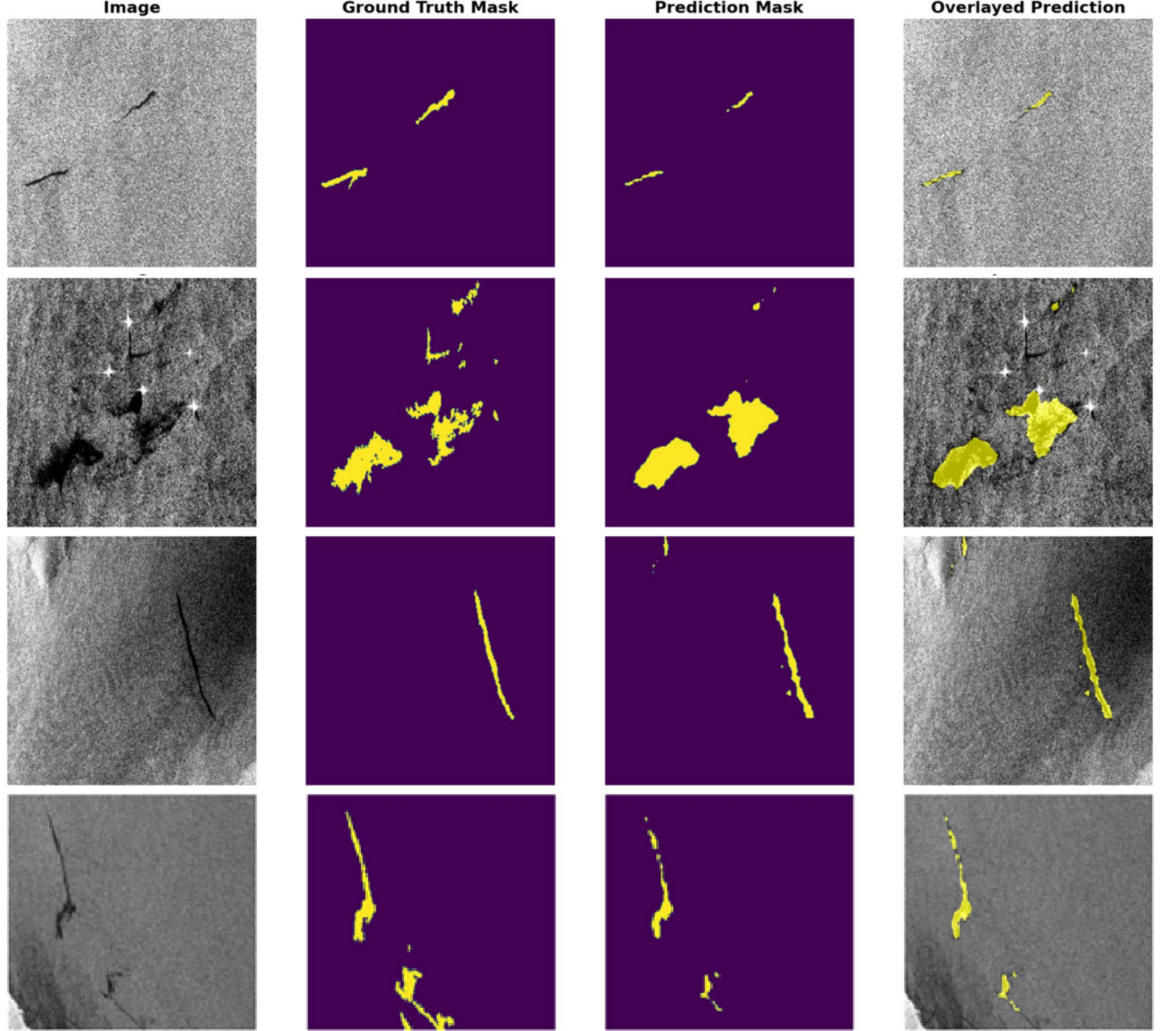
Predictions generated by the Egyptian-data-trained model on its validation data. Data is prepared by Keras-CV library v.0.9.0 https://pypi.org/project/keras-cv/ and plotted by Matplotlib v.3.7.1 https://matplotlib.org/.

The model’s qualitative capabilities were demonstrated through its predictions. (Fig. 13) presents examples of these predictions, highlighting the precision in identifying and segmenting relevant features within the SAR imagery.

Overall, while both models exhibited high performance with their trained data, the EMSA-CSN-data-trained model excelled in terms of accuracy and lower loss values, whereas the Egyptian-data-trained model performed slightly better in terms of the MIoU. The latter model’s higher MIoU suggests that it might be more precise in segmentation tasks, which is crucial for accurately delineating oil spill regions. However, the trade-off between accuracy and segmentation precision is evident, as the EMSA-CSN-data-trained model offers higher generalization and accuracy, making it more suitable for broader applications across different geographic regions. In contrast, the Egyptian-data-trained model’s slightly better MIoU highlights its ability to adapt to the specific characteristics of the local environment, making it a valuable tool for region-specific oil spill detection.

**Fig. 14:**
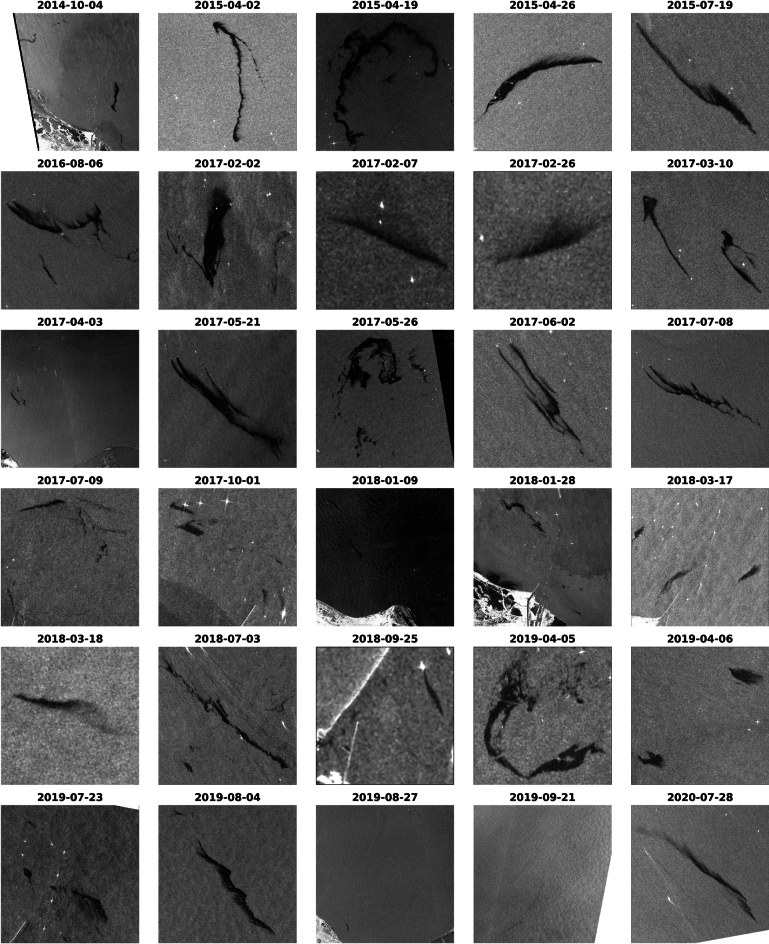
A set of 30 preprocessed testing images. Data is processed by SNAP gpt v.6.0 https://step.esa.int/main/snap-6-0-released/ and plotted by Matplotlib v.3.7.1 https://matplotlib.org/.

For the testing phase, Sentinel-1 SAR images were downloaded, preprocessed, clipped to the specific locations of documented oil spills, and converted to RGB format to be compatible with the input requirements (Fig. 14).

**Fig. 15:**
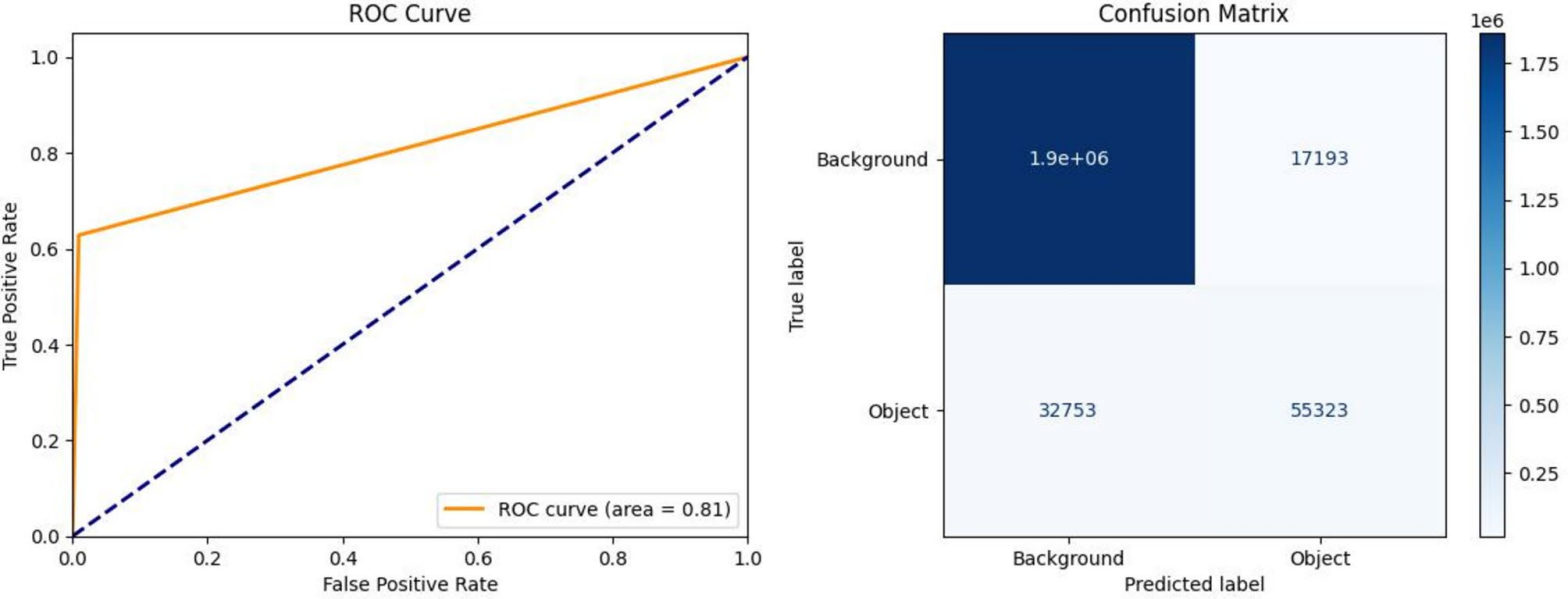
The ROC curve and confusion matrix displaying the performance of the EMSA-CSN-data-trained model on testing data. The presented data is prepared by Scikit-learn library v.1.5.2 https://scikit-learn.org/1.5/whats_new/v1.5.html and plotted by Matplotlib v.3.7.1 https://matplotlib.org/.

**Fig. 16:**
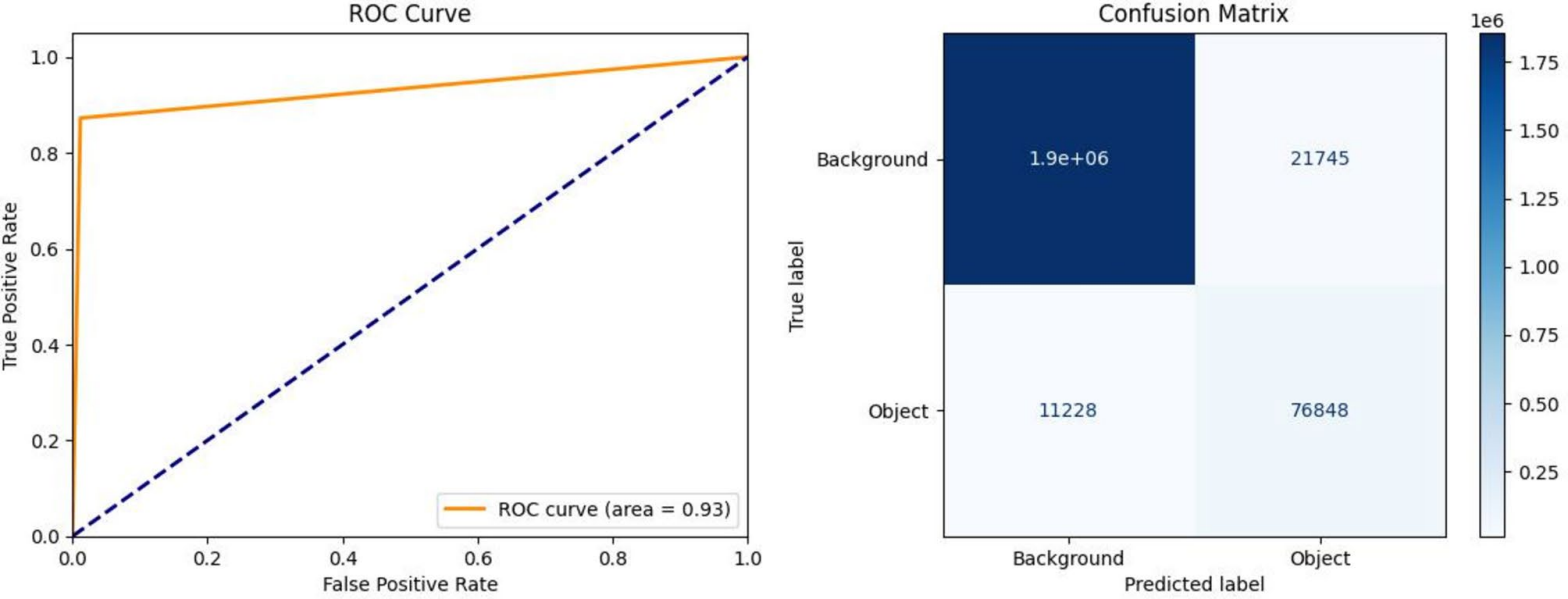
The ROC curve and confusion matrix displaying the performance of the Egyptian-data-trained model on testing data. The presented data is prepared by Scikit-learn library v.1.5.2 https://scikit-learn.org/1.5/whats_new/v1.5.html and plotted by Matplotlib v.3.7.1 https://matplotlib.org/.

While validation data aids in fine-tuning the model training and guiding decisions regarding hyperparameters. Conversely, testing data serves as the ultimate evaluation metric, providing an unbiased assessment of the model’s real-world performance^50–52^. The comparison between the two models reveals notable performance improvements when using the Egypt-data-trained model, as seen in (Figs. 15) and (16). The model trained on EMSA-CSN data achieved a loss of 0.1152, an accuracy of 96.45%, a MIoU of 0.7161, and a ROC area of 0.76. On the other side, the model trained on the Egypt dataset demonstrated superior performance, with a lower loss of 0.0516, a higher accuracy of 98.14%, an improved MIoU of 0.7872, and a significantly higher ROC area of 0.91. Also, confusion matrices show that the EMSA-CSN-data-trained model has a higher false negative rate (missed detections), while the Egyptian-data-trained model detects more actual oil spills correctly. These results indicate that incorporating region-specific data enhances the model’s ability to distinguish oil spills from look-alike features more effectively, reducing false detections and improving segmentation quality.

**Fig. 17:**
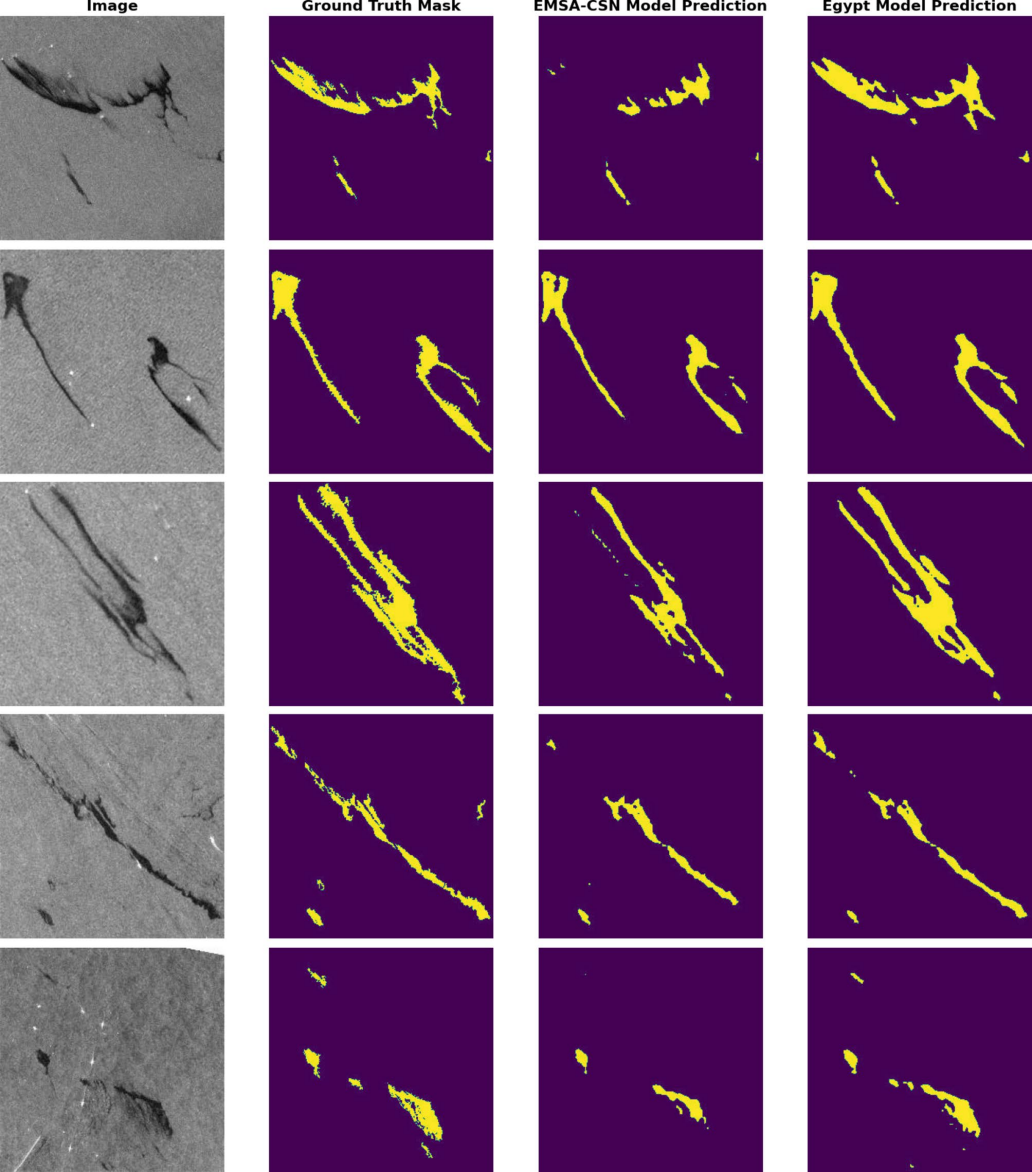
A comparison between predictions generated by EMSA-CSN-data and Egyptian-data-trained models on testing data. The presented data is generated by Keras-CV library v.0.9.0 https://pypi.org/project/keras-cv/ and plotted by Matplotlib v.3.7.1 https://matplotlib.org/.

Both models’ performance is further illustrated through visual representations of prediction results on the testing dataset. (Fig. 17) showcases a selection of sample results from the testing data across the study area to display the enhanced performance of the Egyptian-data-trained model over the EMSA-CSN-data-trained model.

**Fig. 18:**
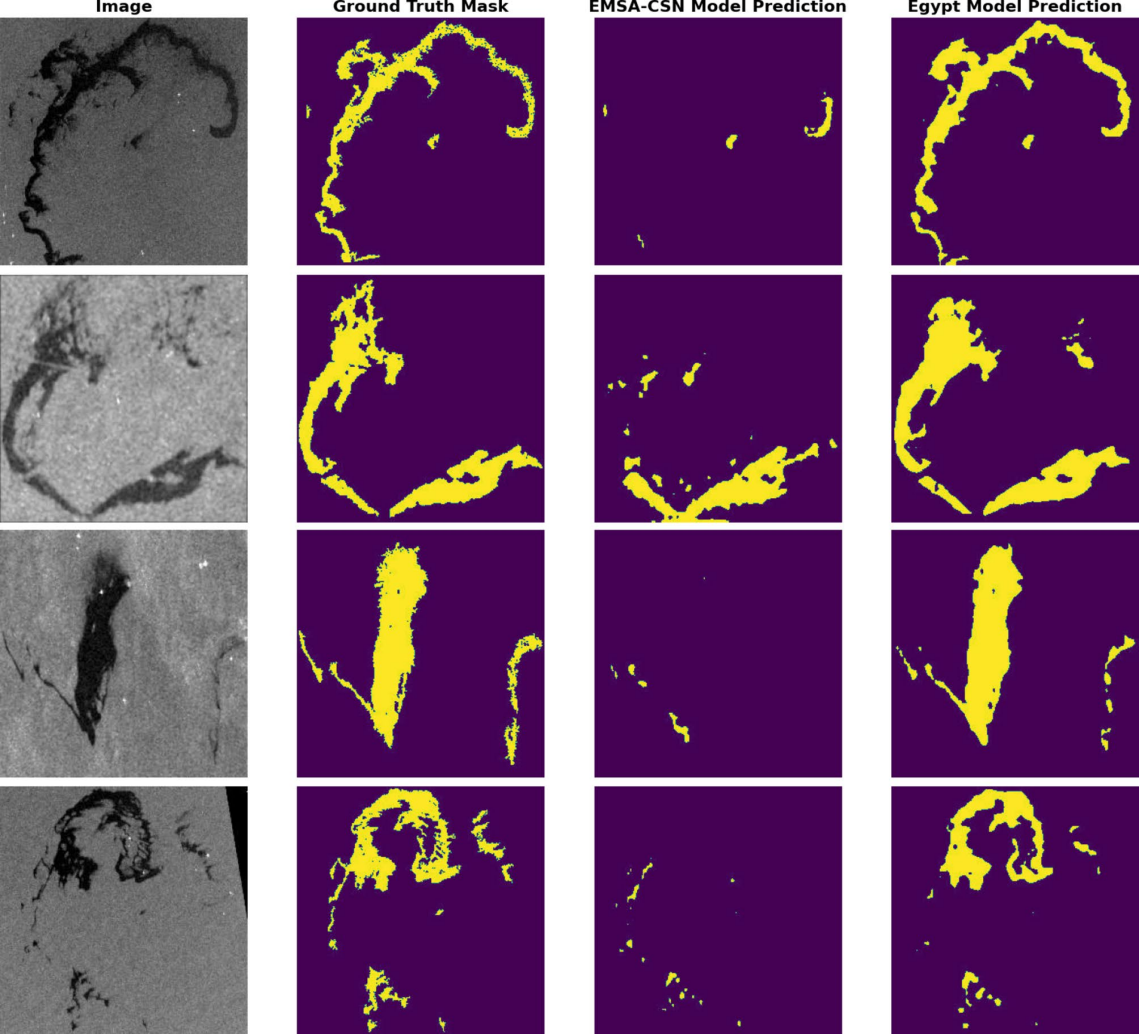
The failure of the EMSA-CSN model to predict significant parts in oil spills while the Egyptian-data-trained models almost replicate the ground truth. The presented data is generated by Keras-CV library v.0.9.0 https://pypi.org/project/keras-cv/ and plotted by Matplotlib v.3.7.1 https://matplotlib.org/.

Notably, several cases reveal that the EMSA-CSN-data-trained model failed to detect significant parts of oil spills, likely due to differences in environmental conditions, oil spill characteristics, and regional variations (Fig. 18).

**Fig. 19:**
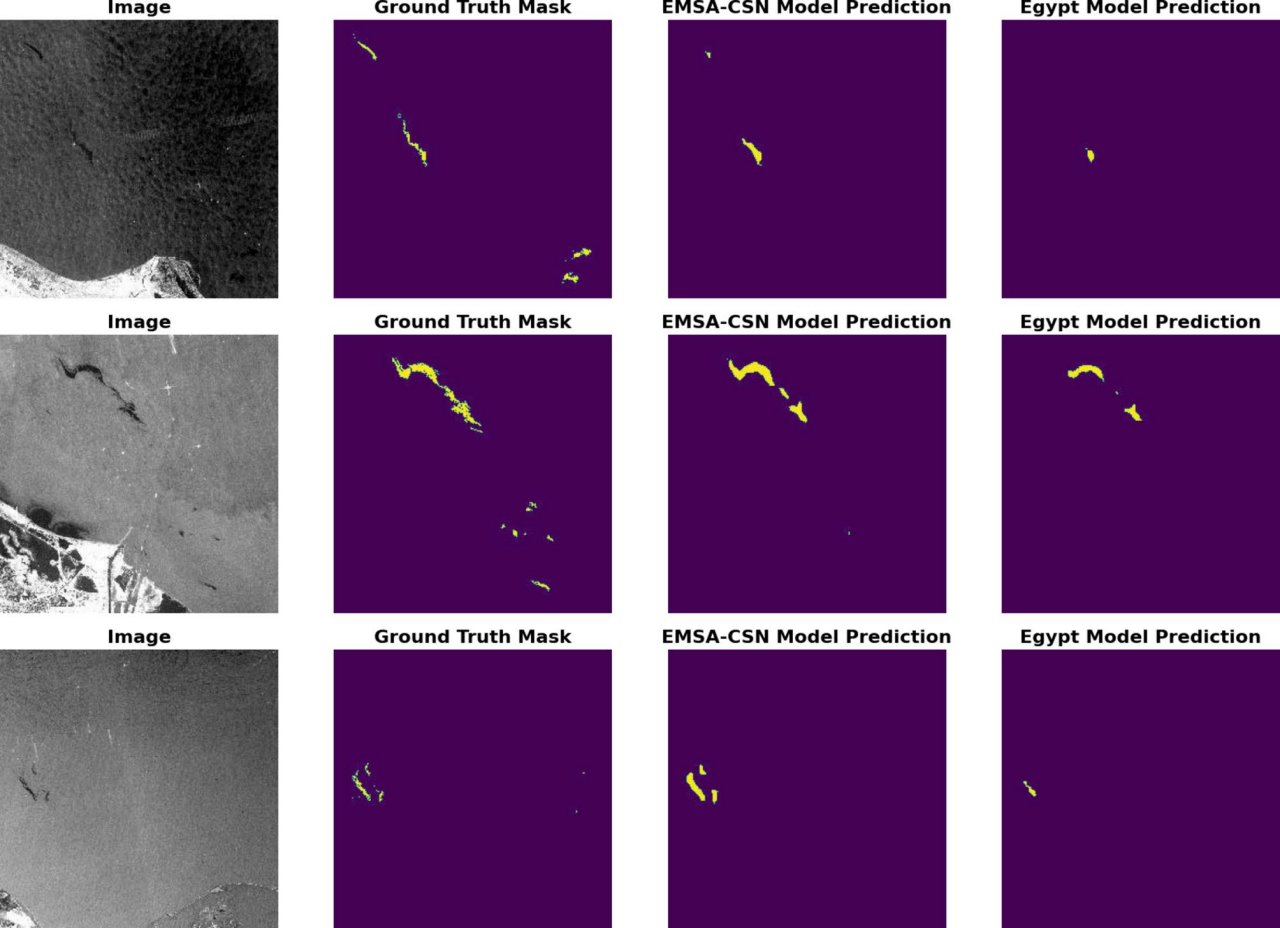
Few Prediction cases where the EMSA-CSN-data-trained model performs better than the Egyptian-data-trained model. The presented data is generated by Keras-CV library v.0.9.0 https://pypi.org/project/keras-cv/ and plotted by Matplotlib v.3.7.1 https://matplotlib.org/.

The EMSA-CSN-data-trained model demonstrated better detection capability in a limited number of cases, primarily when the oil spill is relatively small compared to the overall scene (Fig. 19). This can be attributed to the model’s training on a diverse but generalized dataset, which could be more effective in identifying compact, well-defined spills. In contrast, the Egypt-data model is trained on a localized dataset performed better in detecting larger spill patterns.

**Fig. 20:**
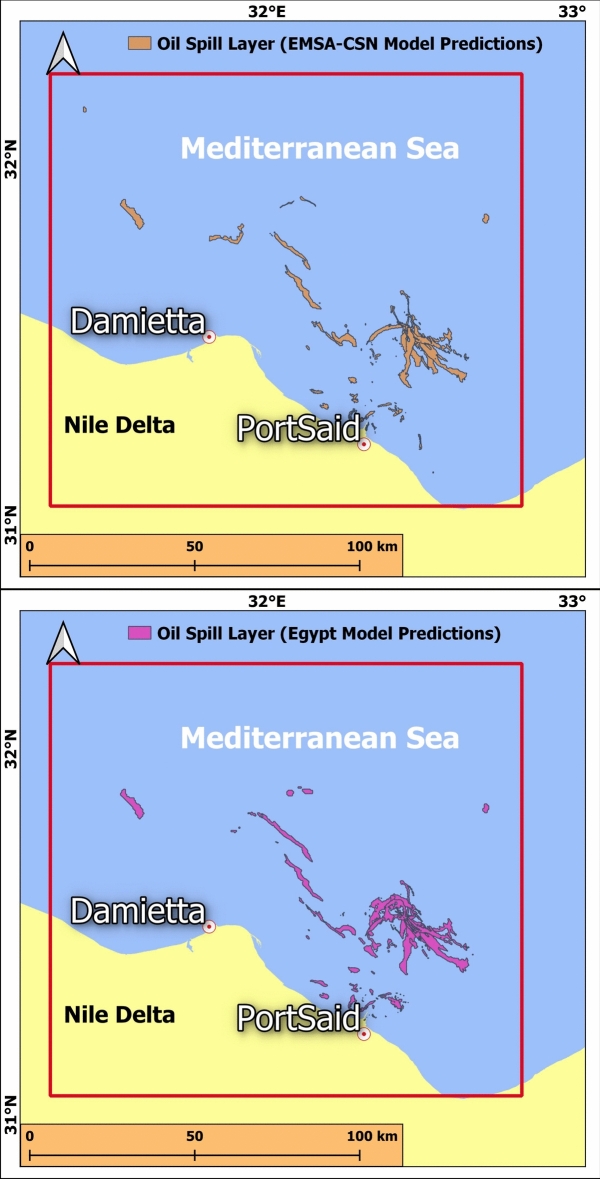
Visual comparison of spatial distribution between EMSA-CSN-data and Egyptian-data-trained models’ predictions. The presented maps are produced by QGIS software v.3.14.1. https://qgis.org/download/.

For a clear understanding of the spatial distribution of oil spills and their respective magnitudes, all the predicted layers are transformed into geolocated images (Fig. 20). By overlaying the detection results onto geographical maps, decision-makers and stakeholders gain valuable insights into the extent and severity of environmental contamination, which aids in a prompt response with the appropriate mitigation efforts.

This analysis reveals that the developed models successfully detect oil spills in the majority of cases, effectively identifying most of the significant spill events. However, certain challenges persist, particularly in the detection of long, narrow oil spills, which the model finds difficult to accurately capture due to their thin profile. Additionally, it is observed that the edges of the detected spills are often less sharp compared to the ground truth data. This lack of precision in edge definition indicates that while the model performs well in recognizing the presence of spills, it may struggle with the fine details, highlighting areas for potential improvement in the model’s segmentation capabilities.

The comparison in Table 3 between the EMSA-CSN-data and the Egyptian-data-trained model presents notable differences in their oil spill prediction accuracy. The Egyptian-data-trained model exhibits a total predicted area of 421.20 km^2^, closely aligning with the ground truth of 425.20 km^2^, while the EMSA-CSN-data-trained model significantly underestimates oil spills with a total predicted area of 323.98 km^2^. The cumulative error for the EMSA-CSN model is − 101.22 km^2^, whereas the Egyptian-data-trained model shows a much smaller total error of − 4.00 km^2^, indicating a higher overall accuracy.

**Table 3:** Quantitative comparison of oil spill area predictions from the EMSA-CSN-data and the Egyptian-data-trained models relative to the ground truth data.

Date	Ground truth area (km^2^)	EMSA-CSN-data trained model predicted area (km^2^)	Egyptian-data trained model predicted area (km^2^)	EMSA-CSN-data trained model error (km^2^)	Egyptian-data trained model error (km^2^)
2014-10-04	42.23	47.126	48.35	4.90	6.12
2015-04-02	4.00	4.404	7.02	0.40	3.02
2015-04-19	24.14	4.972	49.96	-19.16	25.82
2015-04-26	11.32	12.831	14.29	1.51	2.97
2015-07-19	6.22	7.222	8.6	1.00	2.38
2016-08-06	17.59	22.244	29.88	4.66	12.29
2017-02-02	13.00	2.94	13.18	− 10.06	0.18
2017-02-07	1.11	1.894	1.88	0.78	0.77
2017-02-26	2.28	1.368	2.67	− 0.92	0.39
2017-03-10	5.91	5.139	7.57	− 0.77	1.66
2017-04-03	16.72	41.923	4.77	25.21	− 11.95
2017-05-21	39.72	40.765	51.26	1.05	11.54
2017-05-26	28.20	0.023	27.41	− 28.17	− 0.79
2017-06-02	7.83	3.38	8.41	− 4.45	0.58
2017-07-08	17.99	19.819	22.37	1.82	4.38
2017-07-09	5.10	3.66	2.97	− 1.44	− 2.13
2017-10-01	3.71	2.206	2.38	− 1.50	− 1.33
2018-01-09	45.20	19.53	15	− 25.67	− 30.20
2018-01-28	20.72	15.651	16.81	− 5.07	− 3.91
2018-03-17	3.35	5.783	4.97	2.43	1.62
2018-03-18	2.36	2.159	3.68	− 0.20	1.32
2018-07-03	8.63	7.438	10.17	− 1.19	1.54
2018-09-25	0.30	0.837	0.43	0.53	0.13
2019-04-05	4.67	4.787	6.39	0.11	1.72
2019-04-06	4.41	5.506	6.41	1.09	2.00
2019-07-23	7.05	1.783	8.74	− 5.27	1.69
2019-08-04	19.27	20.382	18.89	1.11	− 0.38
2019-08-27	1.83	0.8	0.6	− 1.03	− 1.23
2019-09-21	41.30	3.544	3	− 37.76	− 38.30
2020-07-28	19.04	13.867	23.14	− 5.17	4.10
Total	425.20	323.98	421.20	− 101.22	− 4.00

The key advantage of the Egyptian-data-trained model is its more balanced error distribution, with a total error of − 4.00 km^2^, indicating that its overestimations and underestimations nearly cancel out. In contrast, the EMSA-CSN-date-trained model exhibits a substantial negative error of − 101.22 km^2^, revealing a clear pattern of underestimating the detection area, which can be problematic in real-world applications where detecting the full extent of an oil spill is crucial.

In addition, the EMSA-CSN-data-trained model frequently underestimates larger oil spills. For instance, on April 19, 2015, the ground truth area was 24.14 km^2^, but the model predicted only 4.97 km^2^, significantly underrepresenting the actual spill. In contrast, the Egyptian-data-trained model predicted 49.96 km^2^, which, while an overestimation, still captures a more comprehensive spill extent. Similarly, on September 21, 2019, a large oil spill of 41.3 km^2^ was severely misclassified by the EMSA-CSN-data-trained model, which only detected 3.54 km^2^, whereas the Egyptian-data-trained model’s prediction of 3 km^2^ also fell short but remained within the same range.

While the EMSA-CSN-date-trained model struggles with larger spills, it performs relatively well on smaller spills. For example, on April 2, 2015, a small spill of 4 km^2^ was predicted at 4.4 km^2^ by the EMSA-CSN-date-trained model, which is a close approximation. However, the Egyptian-data-trained model slightly overestimated it at 7 km^2^, showing minor discrepancies. Nonetheless, the Egyptian-data-trained model remains more consistent in predicting oil spills across different sizes, making it a more reliable approach for general detection.

Generally, the EMSA-CSN-date-trained model performs reasonably well in detecting small spills but struggles significantly with larger spills, with a dominant underestimation of their extent. The model trained on the Egyptian localized data provides a much closer match to ground truth measurements, making it a more reliable model for oil spill detection in this study area.

### Study limitations

Despite their promising results, both the EMSA-CSN-data and Egyptian-data-trained models have certain limitations in oil spill detection using SAR imagery. These limitations affect their accuracy, generalizability, and reliability across different scenarios.

The EMSA-CSN-date-trained model has difficulty adapting to local conditions, as it was trained on a broader dataset that may not fully capture the unique oceanographic and environmental characteristics of the Egyptian seas. A major limitation is its poor detection of large oil spills, often underestimating their size, as seen in cases like April 19, 2015, and January 9, 2018. While the EMSA-CSN-date-trained model performs relatively well in detecting smaller spills, its tendency to produce high false negatives suggests challenges in distinguishing oil from look-alike substances.

On the other hand, the Egyptian-data-trained model tends to overestimate oil spill areas, particularly in cases involving mid-sized spills, such as those on April 19, 2015, and May 21, 2017. While the model successfully detects larger spills, it occasionally misclassifies surrounding water or other elements as oil, resulting in false positives. Furthermore, the model’s performance is highly dependent on the size and quality of the training dataset. Although it benefits from being trained on localized data, its accuracy may decline when applied to different imaging conditions.

Moreover, SAR imaging, despite being an effective tool for detecting oil spills, faces several notable limitations. A primary challenge is accurately distinguishing between oil spills and look-alikes, such as seaweed or calm waters, which can appear similar in SAR imagery. Additionally, SAR’s performance is highly sensitive to wind conditions; detection capabilities are disputed in either very calm or very windy conditions due to reduced contrast. The technology cannot also measure oil spill thickness, a critical factor for assessing environmental impact. Sentinel-1’s 10-m spatial resolution limits its ability to capture the detailed morphology of small spills, and its six-day revisit time restricts continuous monitoring capabilities. SAR images are also affected by inherent speckle noise, which complicates identification, particularly in rough sea conditions. Recognizing these limitations is essential for effective oil spill monitoring.

While large Sentinel-1 scenes represent a challenge for the model, which accepts images of size 256 × 256 pixels, an automated workflow is implemented that first clips the larger image into smaller regions and then rasterizes these clipped areas into 256 × 256 pixel tiles. Each tile is processed independently by the model, while the original geospatial coordinates are preserved throughout the workflow. The clipping ensures that all portions of the larger area are considered. Once processed, the individual tile outputs are stitched back together to form a continuous, seamless classification map of the larger area. To maintain spatial accuracy, the geographic coordinates of each tile are retained during both the clipping and rasterization processes.

## Conclusion and future work

In conclusion, the northern entrance of the Suez Canal demands continuous monitoring for oil spills to protect the critically important maritime traffic and avoid the potential environmental risks associated with oil contamination in this strategic waterway. The present study demonstrated the effectiveness of the DeepLabv3 + model in detecting oil spills, achieving high accuracy and appropriate mean intersection over union during both validation and testing phases. The model’s ability to generalize well across different spill scenarios underscores its potential for operational use in oil spill monitoring.

Comparative analysis between the EMSA-CSN-data and the Egyptian-data-trained models revealed that the latter provided improved detection accuracy, particularly for large and medium-sized spills. However, challenges remained in detecting smaller spills, distinguishing oil from look-alike substances, and refining the model’s performance in complex oceanographic conditions. The Egyptian-data-trained model demonstrated greater sensitivity to localized spill patterns but tended to overestimate spill areas in some cases, whereas the EMSA-CSN-date-trained model performed better for smaller spills but underestimated large spills. These findings highlight the importance of region-specific training data and adaptive modeling techniques to improve detection performance.

To improve the model’s performance, future efforts should focus on fine-tuning the architecture by adding convolutional layers to improve detail recognition and adjusting hyperparameters to enhance sensitivity to smaller oil spills. Expanding the training dataset to include a wider variety of spill sizes will enable the model to generalize better across real-world scenarios and improve its ability to generalize across various conditions. Incorporating multi-modal data, such as infrared and multispectral imagery, and in situ measurements can provide complementary information that enhances the model’s detection capabilities. Additionally, exploring advanced architectures like attention mechanisms and utilizing active learning strategies will help the model to learn from mistakes.

## Data Availability

The necessary procedures to generate the data and the methodology have been outlined in the manuscript. The data that support the findings of this study are available with the corresponding author and can be disseminated upon a reasonable request.
